# Light-eye-body axis: exploring the network from retinal illumination to systemic regulation

**DOI:** 10.7150/thno.106589

**Published:** 2025-01-02

**Authors:** Yi Zeng, Rong Rong, Mengling You, Peng Zhu, Jinglin Zhang, Xiaobo Xia

**Affiliations:** 1 Eye Center of Xiangya Hospital, Central South University, Changsha, Hunan, 410008, P.R. China.; 2 Hunan Key Laboratory of Ophthalmology, Changsha, Hunan, 410008, P.R. China.; 3 National clinical key specialty of ophthalmology, Changsha, Hunan, 410008, P.R. China.; 4 National Clinical Research Center for Geriatric Diseases (Xiangya Hospital), Central South University, Changsha, Hunan, 410008, P.R. China.

**Keywords:** light, retina, ipRGCs, systemic regulation, organ interaction

## Abstract

The human body is an intricate system, where diverse and complex signaling among different organs sustains physiological activities. The eye, as a primary organ for information acquisition, not only plays a crucial role in visual perception but also, as increasing evidence suggests, exerts a broad influence on the entire body through complex circuits upon receiving light signals which is called non-image-forming vision. However, the extent and mechanisms of light's impact on the body through the eyes remain insufficiently explored. There is also a dearth of comprehensive reviews elucidating the intricate interplay between light, the eye, and the systemic connections to the entire body. Herein, we propose the concept of the light-eye-body axis to systematically encapsulate the extensive non-image-forming effects of light signals received by the retina on the entire body. We reviewed the visual-neural structure basis of the light-eye-body axis, summarized the mechanism by which the eyes regulate the whole body and the current research status and challenges within the physiological and pathological processes involved in the light-eye-body axis. Future research should aim to expand the influence of the light-eye-body axis and explore its deeper mechanisms. Understanding and investigating the light-eye-body axis will contribute to improving lighting conditions to optimize health and guide the establishment of phototherapy standards in clinical practice.

## Background

The eye, as the primary organ for information acquisition in the human body, serves as a bridge for communicating the external world. Besides its fundamental functions of visual information acquisition and perception of day-night changes, recent years have seen reports on the eye's influence on other bodily functions or disease progression in non-image vision, which is an intriguing discovery 1,2. In previous research and focus, we understand that the eye is a barometer of overall health, with many systemic diseases affecting the eye and presenting significant clinical manifestations 3,4. However, reports on the eye's impact on overall bodily functions and specific regulatory mechanisms are relatively scarce, which is crucial for the study of organ interaction in the body.

In our bodies, not only do different organs perform their respective duties, but there is also close communication between organs, working together to maintain homeostasis. These organ communication networks play a vital role in the human body every day. Clarifying the modes and mechanisms of inter-organ communication will help understand the mechanisms of local lesion development from a holistic perspective. It is known that organs often secrete various factors that circulate to another organ for dialogue, exerting corresponding biological functions through these active factors and affecting organ operation, such as the hypothalamic-pituitary-thyroid axis. The eye, as a relatively special organ in the body, has multiple ocular barriers, making it a relatively independent system. However, many small molecule proteins, peptides, and factors can freely shuttle through the blood-retinal barrier, thereby achieving information exchange 5. But compared to transmitting information through the circulatory system, the eye, due to its unique optical system and physiological functions, is more likely to receive optical signal and transmit neuron signal to achieve multi-organ dialogue.

Based on this, how the eye regulates and affects other organs and the physiological and pathological changes of the body, answering these questions will help us understand more comprehensively the key link of the eye in the operation of the complex factory of the body, and it is of great significance for the systematic analysis of the occurrence and development of other diseases. In this paper, we propose the concept of the light-eye-body axis to systematically encapsulate the extensive non-image-forming effects of light signals received by the retina on the entire body. This article will sort out and summarize the frontier content in this area, and discuss and look forward to the related uninvolved fields, striving to provide possible development directions and ideas for research in this field.

## 1. Photographic structure foundation of the light-eye-body axis

Light, the genesis of all life, has been profoundly influential throughout the evolution of our planet. With the advent of industrial civilization, the impact of artificial light on biological organisms has become increasinggly significant 6. Understanding how organisms perceive light and how light signals regulate organisms holds significant scientific and practical value. For image-forming vision, light carries information from the external world, which after passing through the refractive system of the eye (such as the lens and vitreous body), and is finally projected onto the retina. Upon reaching the retina, light signals are first received by the rod and cone cells, then relayed to the first-level neurons, the bipolar cells, and subsequently to the second-level neurons, the retinal ganglion cells (RGCs) which are the sole neuronal intermediaries that bridge the retina with the brain, facilitating the transmission of visual information from the ocular periphery to the central nervous system. The axons of the ganglion cells converge to form the optic nerve, which, after passing through the lateral geniculate body of the third-level neurons, forms the optic radiation projecting to the visual cortex, thus forming vision. The aforementioned retinal circuit forms the structural basis for the image-forming vision. Non-image-forming vision and image-forming vision utilize similar retinal circuits, yet there are distinctions in the photoreceptor signals, and the brain regions they regulate 7.

In the realm of non-image-forming vision, three types of photoreceptor cells have been identified that play a pivotal role in detecting light and initiating a variety of physiological and behavioral responses. These cells include the well-known rod and cone photoreceptors, and an additional type known as the intrinsically photosensitive retinal ganglion cells (ipRGCs). Rod cells are extremely sensitive to light, and even very weak light can activate them 8. That's why we can see clearly in dark environments. In contrast, cone cells, while less sensitive to light, play a crucial role in forming color vision. Rod and cone cells are important photoreceptor cells, primarily forming brightness, color, and visual effects. In addition to these, there is another type of photoreceptor cell in the retinal ganglion cells, discovered in 2002, named ipRGCs. IpRGCs express the photopigment melanopsin, endowing them with the ability to sense light directly 9. This unique feature distinguishes ipRGCs from other photoreceptors, which are primarily involved in image-forming vision. The expression of melanopsin in ipRGCs is crucial for a variety of light-sensitive functions that contribute to the regulation of series of physiological processes such as circadian rhythms, pupil constriction, and even mood and cognitive processes 10. Upon discovery, they were named one of the “Top Ten Scientific Breakthroughs” of 2002 by Science magazine, underscoring their importance. Due to the significant role of ipRGCs in the regulation of light-eye-body axis, they have been the focus of extensive research. In the forthcoming sections, a detailed exploration of these cells will be presented.

### 1.1. IpRGCs

RGCs are the only neurons in the retina that project to the brain and transmit retinal information from the eye to the brain. To date, transcriptomics studies have identified more than 40 subtypes of RGCs 11. Several major types contained directionally selective ganglion cells which stand out for their optimal response to stimuli moving in a specific direction, exemplifying the retina's ability to discern and convey directional motion to the brain 12; the alpha RGCs, as the primary conduits for visual signals, are pivotal in the transmission of visual information to the cerebral cortex, serving as the sentinels of sight 13; the J-RGCs, identifiable by their expression of the junctional adhesion molecule B, exhibit a unique selectivity for stimuli moving from the soma to the dendritic direction, effectively detecting upward motion within the visual field 14.

**Figure 1:**
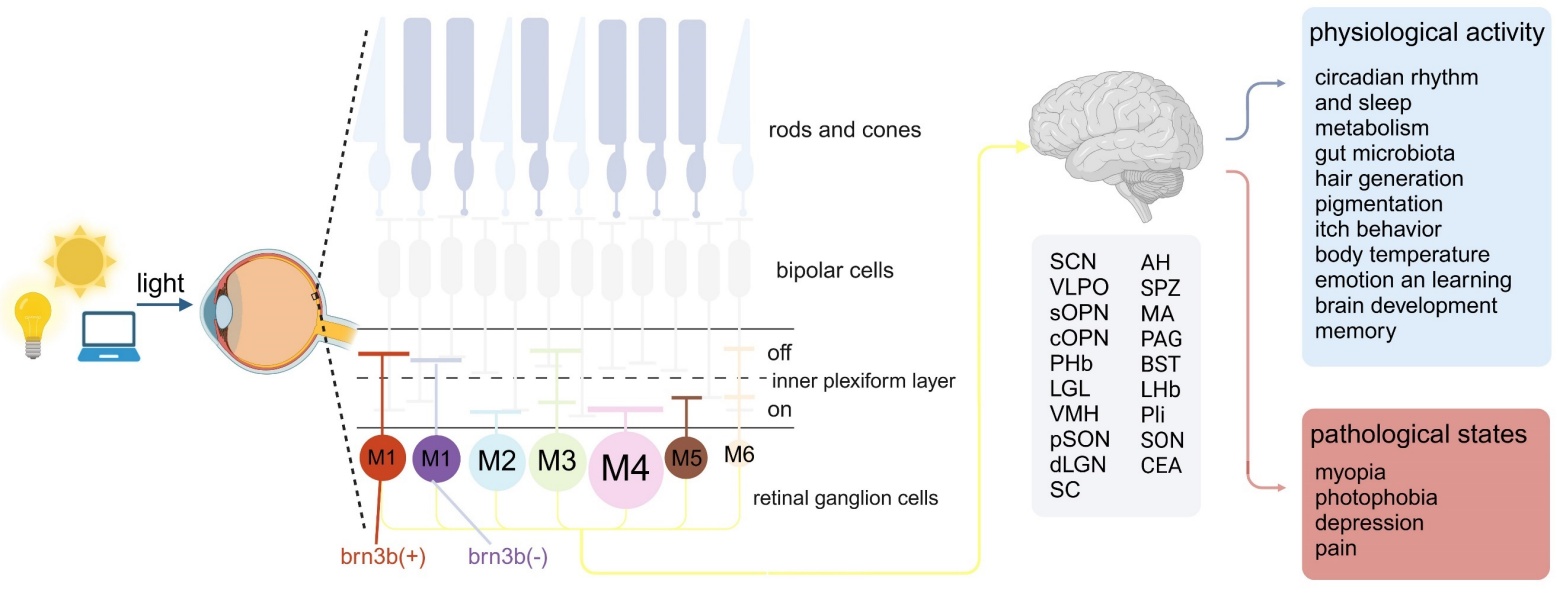
** Light signals entering the retina are transmitted to various brain regions by intrinsically photosensitive retinal ganglion cells (ipRGCs), exerting extensive effects on the organism.** Upon penetrating the eye, light is projected onto the retina, where it is captured by ipRGCs. These ipRGCs are categorized into six distinct subtypes, each distinguishable by the size of their soma, the dendritic arborization within the retinal IPL, and their projection sites within the brain. Following the reception of light signals, ipRGCs project to downstream brain areas such as the SCN, VLPO and IGL, thereby broadly modulating the organism's physiological functions and pathological conditions. ipRGCs: intrinsically photosensitive Retinal Ganglion Cells; IPL: inner plexiform layer; SCN: suprachiasmatic nucleus; VLPO: Ventrolateral Preoptic Nucleus; Suprachiasmatic Nucleus; IGL: Intergeniculate Leaflet; sOPN: olivary pretectal nucleus shell; cOPN: olivary pretectal nucleus core; PPN: posterior pretectal nucleus; PHb: perihabenular nucleus; vLGN: ventral lateral geniculate nucleus; VMH: ventromedial nucleus; pSON: perisupraoptic nucleus; dLGN: dorsal lateral geniculate nucleus; SC: superior colliculus; AH: anterior hypothalamus; SPZ: subparaventricular zone; MA: medial amygdala nucleus; PAG: periaqueductal gray; BST: bed nucleus of the stria terminalis; LH: lateral hypothalamic area; LHb: lateral habenula; PLi: posterior limitans; SON: supraoptic nucleus; CEA: central amygdala.

IpRGCs differ from other RGCs, in addition to receiving signal input from photoreceptor cells, they can directly respond to light exposure by expressing the photopigment melanopsin 15. IpRGCs have a high threshold of activation, prolonged response latency, and a sluggish return to baseline. These physiological traits endow ipRGCs with the capacity to integrate light information over an extended period of illumination, thereby functioning as effective irradiance detectors 15. Moreover, the single-photon responses of melanopsin clearly demonstrate that this photopigment is at least as sensitive as the classical rod and cone photopigments 16. Due to the presence of their characteristic molecule, melanopsin, ipRGCs possess the dual ability of receiving light signal input from cone and rod cells and projecting to dozens of brain areas, as well as the ability to respond directly to light through melanopsin, thus serving as a critical bridge in light-eye-body axis (Figure 1).

Since the discovery of melanopsin, research has revealed six subtypes of melanopsin ganglion cells (M1-M6), each with a unique set of morphology, distribution, connections, physiology, projections, and functions. These subtypes can mainly be distinguished by morphological characteristics, the level of dendritic stratification in the inner plexiform layer (IPL) of the retina, and their dendritic size and complexity. According to the morphology of the axon terminals of bipolar cells and their distribution in the inner plexiform layer, the IPL can be divided into OFF sublayers and ON sublayers. When the light is enhanced, ON layer bipolar cells show depolarization, while OFF layer bipolar cells show hyperpolarization 17. The dendrites of M1 ipRGCs are distributed in the OFF sublayer of the IPL, and among all ipRGC subtypes, their soma is relatively small and has a relatively small and simple dendritic structure 18. As the first discovered type of melanopsin expressed RGC, they showed strong melanopsin immunoreactivity, and it is easy to stain them with traditional melanopsin immunohistochemistry 19. What is special is that there are two subgroups of M1 ipRGCs, which are mainly distinguished according to whether they express the transcription factor Brn3b, and the axons of these two subgroups also project to different areas of the brain, thus they have different functions 20. At the same time, M2-M6 type ipRGCs also express Brn3b. M2 ipRGCs have dendrites stratified in the ON sublayer, compared to M1 ipRGCs, their soma is larger and has a more complex dendritic structure 21. M3 ipRGCs are similar to M2 cells in terms of soma size and dendritic tree size and complexity, but M3 ipRGCs' dendrites are distributed in both the ON and OFF layers of the IPL 22,23. Among all ipRGCs, M4 ipRGCs have the largest soma and dendritic structure, distributed in the ON-IPL sublayer, but their melanopsin expression is the lowest 24,25. The discovery of M5 ipRGCs is based on a more sensitive Cre-based melanopsin detection system because the melanopsin immunoreactivity of M5 ipRGCs is very low 24. Distributed in the ON sublayer, M5 ipRGCs cells have a relatively small soma and highly branched dendritic structure 26. M6 ipRGCs are the latest discovered type of ipRGCs, and they have the smallest cell volume and the densest dendritic structure, with an average of about 100 branches. Similar to M3 ipRGCs, they have dendritic stratification in both the ON and OFF sublayers of the IPL 27.

In addition to having different morphological characteristics, different ipRGCs also have different functions, which are mainly determined by their projections to different areas of the brain. The projections of M1 ipRGCs are the most extensive, with many targets in the midbrain, thalamus, and hypothalamus 20. Brn3b negative M1 ipRGCs mainly project to suprachiasmatic nucleus (SCN), regulating circadian rhythm light induction. While Brn3b positive M1 ipRGCs have a very wide range of projections, they can project to non-imaging areas such as lateral habenula (LHb), perisupraoptic nucleus (pSON) and ventromedial nucleus (VMH), linking the regulation of emotions, body temperature and eating with brain light-induced behavior 28,29. M2 ipRGCs also project to multiple areas of the brain, giving it the potential to function in both image-forming (superior colliculus (SC) and dorsal geniculate nucleus (dLGN) and non-image-forming vision (SCN and olivary pretectal nucleus (OPN)) 24,30. Due to the sparsity of M3 cells, it is currently difficult to determine their axon targets in the brain 23. Although there is evidence that M3 ipRGCs may project to SC, little is known about the function of this type, which may be because this subtype has not been widely studied 31. M4-M6 cells are consistent in function, their axons all project to visual-related brain areas, such as dLGN and the SC 26,27,31.

### 1.2. Rods and cones

Are rod and cone photoreceptors implicated in non-image-forming vision? There is evidence that ipRGCs receive light signals transmitted from these classic photoreceptors. This interaction significantly broadens the dynamic range and spectral sensitivity of the retinal input to ipRGCs 32. Melanopsin, the photopigment expressed in ipRGCs, exhibits peak sensitivity to blue light around 480 nm 33, whereas rods and cones offer a complementary sensitivity to shorter, medium, and longer wavelengths, as well as under low-light conditions 34. Trichromatic primates have three cone types - long (L), medium (M) and short (S)-wavelength sensitive, enhancing the spectral breadth of light input available for non-image forming vision. In dim light, rods primarily detect light, and the resultant light-induced responses can activate ipRGCs 34.

The involvement of rod and cone photoreceptors in non-image forming vision is further supported by studies utilizing melanopsin knockout or rod/cone knockout models, which have demonstrated the collective contribution of all three photoreceptor types to circadian photoentrainment 35. Moreover, recent research has indicated that rod and cone photoreceptor input can modulate ocular development, thereby influencing myopia 36.

### 1.3. Bipolar cells

Bipolar cells serve as the conduit for signal transmission from photoreceptor cells to ipRGCs, playing a pivotal role in the light-eye-body axis. The M1 cells, initially perceived as enigmatic, exhibit sustained ON responses to light stimuli, yet they are stratified in the OFF sublamina of the IPL, a seeming contradiction to their functional classification 15,21. Interestingly, M1 cells deviate from the traditional IPL stratification, forming en passant synapses with ON bipolar cells within the OFF sublamina, a structural anomaly that challenges our understanding of retinal organization 37. In contrast, M2 cells, stratifying in the ON sublamina of the IPL, respond to light onset without relying on the ectopic synapses characteristic of M1 cells 15. Instead, they engage in a synaptic triad with ON cone bipolar cells and inhibitory monostratified amacrine cells, which are positioned in the S4-5 plexus of the IPL 38. M3 cells, similar to M2 in their stratification, predominantly rely on extrinsic synaptic input from the ON cone pathway for their light response. Although it is hypothesized that M3 cells, like M1, may form en passant synapses with ON bipolar cells in the OFF sublamina, this connection has not yet been conclusively demonstrated 37. M4 and M5 cells both receive input from ON cone bipolar cells 39. The bistratified M6 cells, despite their potential to act as ON-OFF cells responding to both light onset and offset, are primarily driven by extrinsic synaptic input from the ON pathway, with an intrinsic melanopsin response that is notably weak 27. This intricate interplay of cellular responses and synaptic connections within the light-eye-body axis underscores the complexity of visual signal processing and allowed for a wide range of regulatory effects (Figure 1).

### 1.4. What's next after photoreception

How did rod, cone and melanopsin photoresponses integrate? Studies in rodents have demonstrated that specific ablation of ipRGCs results in a near-total loss of non-image forming responses, while image forming responses remain largely intact 40. This suggests that the photic signals initiated by rods and cones for NIF responses are primarily transmitted through ipRGCs, establishing these cells as the principal nodal point for integrating the photoresponses of all three photopigment systems. Furthermore, due to their expression of melanopsin, ipRGCs serve as the sole conduit for NIF responses.

However, as research deepened, the limitations of this perspective have become apparent. Firstly, it remains unclear whether the expression of melanopsin in ipRGCs affects their role in mediating rod/cone-initiated responses. For instance, does melanopsin simply add to the outer retinal response, or does it enhance the rod/cone-initiated response in some way? Secondly, there are multiple subtypes ipRGCs, with certain subtypes, such as the M4 type, capable of projecting to brain areas related to image formation, thus impacting visual perception. Additionally, the photic signals initiated by rods and cones for NIF vision are not entirely transported by ipRGCs; they can also be carried by non-photoreceptive RGCs 41. Lastly, beyond neural transmission, photoreceptor cells can also influence the organism through paracrine or immune pathways, which are often overlooked in many studies.

Based on these discussions, we proposed the concept of the "light-eye-body axis" to systematically summarize the broad non-image forming effects of light signals received by the retina on the entire body. The following sections will provide a detailed introduction to how the light-eye-body axis affects the organism, its specific mechanisms, and applications for improving health.

## 2. The role of light signals through the eyes in regulating physiological changes throughout the body

**Figure 2:**
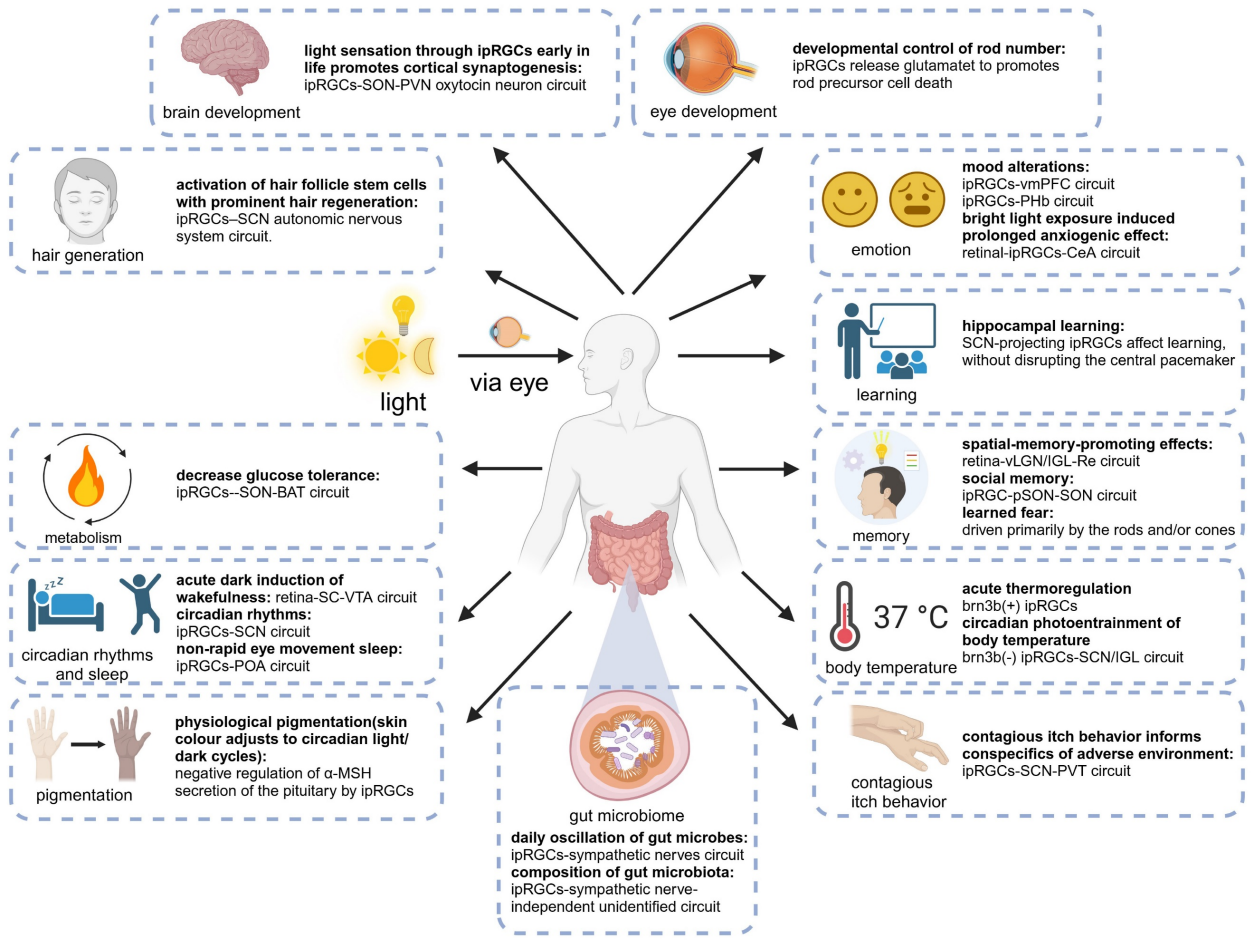
** The light-eye-body axis exerts a widespread impact on the organism's physiological functions.** The light-eye-body axis affects bodily functions through a wide range of pathways. IpRGCs: intrinsically photosensitive Retinal Ganglion Cells; SON: supraoptic nucleus; PVN: paraventricular nucleus; SCN: suprachiasmatic nucleus; vmPFC: ventromedial prefrontal cortex; PHb: perihabenular nucleus; CeA: central amygdala; vLGN: ventral geniculate nucleus; IGL: intergeniculate leaflet; Re: reunion nucleus; pSON: perisupraoptic nucleus; BAT: brown adipose tissue; VTA: ventral tegmental area; POA: preoptic area; α-MSH: alpha-melanocyte-stimulating hormone; PVT: paraventricular nucleus of the thalamus.

Numerous fundamental physiological functions of the human body are regulated by light signals, which was substantiated by extensive animal experiments and clinical studies 42,43. With the deepening of research, the light-eye-body axis has been identified as playing a pivotal role in this regulatory mechanism (Figure 2). As the quest for quality of life and the understanding of light quality continue to ascend, elucidating the scope of influence and the underlying pathways of the light-eye-body axis becomes increasingly vital. In this section, we delve into how light, through the light-eye-body axis, exerts its influence on a multitude of physiological functions across the body.

### 2.1. Circadian rhythm and sleep

In this industrialized society, artificial light at night is ubiquitous and severely disrupts human sleep. The impact of light on sleep is multifaceted, indirectly through the light entrainment of circadian rhythms and directly through acute mechanisms 44. As awareness of the quality of light in daily life increases, elucidating the impact of light on sleep and the underlying neural circuit mechanisms is crucial for human health and society. In this section, we will explore the chronic effects of light through the eyes on circadian rhythms and the acute effects on sleep.

Initially, it was widely believed that the circadian center of the SCN was regulated by light signals from the eyes, independent of rod and cone photoreceptors 45. These observations also facilitated the discovery of ipRGCs, which are themselves photosensitive and project to the SCN 33. Strong anatomical evidence also shows how ipRGCs project to the brain and affect the circadian center 46. Although ipRGCs have been recognized as the sole input to the SCN, it is evident that in addition to ipRGCs, rods and cones play a crucial role in regulating photic synchronization of circadian rhythms. As the removal of the melanopsin gene results in the disappearance of ipRGCs' light response, but the photic synchronization of circadian rhythms is largely unaffected. And the triple knockout of rod, cone, and ipRGC cells leads to the disappearance of photic synchronization 47. Considering the relatively insensitive phototransduction of melanopsin in ipRGCs, which cannot drive physiological responses at low light intensities, the supplementation of rod and cone photoreceptor signals is necessary for the integrity of circadian photic synchronization 48.

The relative contribution of these three photoreceptor cells to circadian photic induction has been controversial. By studying the circadian functions of rod cells, cone cells, and ipRGCs separately, Altimus *et al.* found that rod photoreceptors play a key role in circadian photic induction at light intensities ranging from at least 0.1 lux to 10 lux. The knockout of rod cells in mice leads to the disappearance of circadian photic synchronization at scotopic light levels, indicating that rod cells are necessary for circadian photic synchronization at low light intensities and are mediated by the rod-bipolar pathway. However, at high light intensities and long durations of illumination, it is mediated by the rod-cone pathway and the intrinsic photosensitivity of ipRGCs 49.

Analyzing the above conclusions, we find that the three photoreceptor cells are sufficient conditions for activating circadian photic induction, but a key question remains unresolved: is the photic signal for circadian induction transmitted by ipRGCs or by other types of RGCs? This question was answered by the specific ablation of ipRGCs, as circadian photic induction completely disappeared after ipRGCs 50. Therefore, we can conclude that melanopsin/rod/cone signals are the primary sources of light signals regulating circadian photic induction through the eyes, and ipRGCs play a dual important role in both collecting light signals and serving as an intermediate bridge because they can transmit these light signals to the brain's SCN and produce melanopsin.

Compared to the well-studied impact of light mediated through the eyes on circadian rhythms, the role of the eyes in acute light responses remains largely a mystery. Although studies have shown that light pulses induce sleep and dark pulses induce wakefulness, and that pathways based on rod cells and melanopsin are necessary for regulating the impact of light and dark on sleep 43,51. But the specific pathways have only been reported in recent years. For the wakefulness effect induced by dark pulses, Professor Huang Zhili's group found in mice that the acute dark-mediated wakefulness effect completely disappeared after specific destruction of SC GABAergic neurons or ventral tegmental area (VTA) dopaminergic neurons, revealing that these two types of neurons play an indispensable role in the acute dark-mediated wakefulness effect. RGCs can directly regulate the activity of SC GABAergic neurons and form monosynaptic connections with them, then GABAergic neurons in the SC directly functionally dominate VTA dopaminergic neurons through monosynaptic connections. Their findings demonstrated that during the light phase, a sudden dark pulse diminished the activity of GABAergic neurons located in the SC. This reduction, in turn, led to the disinhibition of VTA dopaminergic cells, which are functionally connected to the SC GABAergic neurons. Their results revealed for the first time the key role of the retinal-SC GABAergic-VTA dopaminergic circuit in the acute dark-induced wakefulness effect in mice 52. For the sleep-inducing effect of light pulses, Samer Hattar's research team found that activating ipRGCs projecting to the preoptic area (POA) increases non-rapid eye movement (NREM) sleep without affecting rapid eye movement sleep. It is well known that the POA is crucial for sleep regulation and is the main sleep center 53. This pathway inputs light signals to the POA neuron subgroup that promotes corticotropin-releasing hormone release, and these POA neurons act by inhibiting tuberomammillary (TMN), lateral hypothalamus (LH), VTA, and dorsal raphe nucleus (DRN), which are regions that mainly promote wakefulness 54,55. In this study, they discovered an ipRGCs-POA- TMN/LH/VTA/DRN neural circuit that is both necessary and sufficient for the acute impact of light on NREM sleep 56.

In summary, the impact of light on sleep can play a complex role through three different pathways, but whether the light signal is from rod/cone photoreceptors or melanopsin, they all cannot bypass the important mediator ipRGCs, which collect light signals and project to downstream brain regions to affect sleep. The three proven pathways are the ipRGCs-SCN for circadian photic synchronization, the ipRGCs-POA for the acute impact on NREM sleep, and the RGCs-SC-VTA for the acute impact on wakefulness. It is worth noting that in wild-type animals, acute light exposure simultaneously affects NREM and rapid eye movement (REM) sleep 57. Although existing research has only proven that acute light exposure can affect NREM through ipRGCs without affecting REM, this may imply that there is still an undiscovered ipRGCs pathway, independent of the three discovered pathways, that has an acute impact on REM sleep.

In this part, we need to pay attention to the fact that the impact of circadian rhythms on overall health and disease has received widespread attention and reporting, but on the one hand, circadian rhythms are induced by light through the eyes, and on the other hand, in the subsequent content, we focus on the direct regulation of the operation of the whole body organ system by light through the eyes, rather than the impact produced by circadian rhythms, highlighting the important hub function of the eyes.

### 2.2. Glucose metabolism

On our planet, light is not only a vital source of life but also one of the primary sources of vision, a crucial sense for living organisms 58. Moreover, the ability of life forms to adjust their metabolic balance of nutrients according to external environmental conditions is an essential survival mechanism, with metabolic disorders often leading to severe diseases. Among these, glucose metabolism is a key component for the normal functioning of life. Through the course of survival and evolution, mammals have developed a precise and complex regulatory network to continuously monitor and dynamically control glucose metabolism 59. Public health surveys have shown that artificial light at night significantly increases the risk of metabolic diseases such as obesity and diabetes 60. Animal experiments also suggest that altering light exposure to regulate circadian rhythms can impact carbohydrate metabolism 61. Thus, the question arises: does light, as one of the most critical external environmental factors, directly regulate glucose metabolism? The involved photoreceptive cells, neural circuits, and peripheral target organs in this process remain unanswered.

Recent research has discovered that adjusting light intensity alleviates glucose metabolism disorders in mice with circadian rhythm disorders, suggesting that light may directly regulate carbohydrate metabolism 62. The findings of Professor Xue Tian's team offer an exhilarating perspective, demonstrating that light can drastically reduce glucose tolerance in mice by activating ipRGCs that govern the supraoptic nucleus (SON). Vasopressin neurons in the SON project to the paraventricular nucleus and then to the GABAergic neurons in the solitary tract nucleus, blocking the β3-adrenergic signaling in brown adipose tissue (BAT) and reducing adaptive thermogenesis, leading to a decrease in GT 2.

Previous studies posited that light might regulate glucose metabolism through the SCN, as disruptions in the SCN's circadian rhythm center affect metabolism, and ipRGCs can project to the SCN, thereby governing circadian rhythms 29. Professor Xue Tian's team has corrected this misconception, pointing out that light regulates carbohydrate metabolism through the SON rather than the SCN.

It is noteworthy that this work not only systematically answers the biological mechanism behind light regulation of blood sugar metabolism in mouse models but also finds the same phenomenon in human trials, indicating that light regulation of blood sugar metabolism may be widespread among mammals. Interestingly, in Professor Xue Tian's research model, blue light was most effective in activating ipRGCs and thus inhibiting BAT thermogenesis. This may explain why cool and warm lights are not merely psychological effects but may have a physiological basis. In our daily life, short-wave light (blue light) feels cool, while long-wave light (red light) feels warm, hence their definition as cool and warm lights 63. The sensation of coldness we experience under blue light stimulation may be a genuine perception resulting from suppressed fat thermogenesis. Therefore, this light-regulated pathway of fat tissue activity may be the physiological foundation for the psychological perception of cool and warm lights.

In this industrial age, exposure to excessive artificial light can significantly increase the metabolic burden 64. Although circadian rhythms cause lower carbohydrate metabolism capacity at night compared to daytime, the results indicate that light-induced suppression of carbohydrate metabolism is independent of circadian rhythm phases and further impairs metabolism 2,65. Thus, under conditions of nighttime light exposure, human blood sugar metabolism capacity is at its worst. These conclusions also suggest that modern humans should pay attention to the health of their light environment, especially concerning nighttime light pollution. Additionally, we should focus on the wavelength, intensity, and duration of artificial light at night and its impact on health.

### 2.3. Emotion and learning

In addition to its visual effects, light possesses a range of potent biological impacts, including the modulation of emotions and learning 66. With the acceleration of industrialization, the influence of various electronic devices on the human body cannot be overlooked. The impact of artificial lighting on mental health is increasingly supported by evidence, and elucidating the causative pathways, specifically the mediating circuits, provides a solid theoretical foundation for raising awareness and implementing effective interventions.

The mediators of light's influence on emotion and cognitive functions have been well-established: ipRGCs 67. Fernandez and colleagues revealed the direct effects of light on learning and emotions utilizing distinct ipRGCs circuits. ipRGCs projecting to the SCN mediate light's impact on learning, independent of the SCN's role in coordinating peripheral biological clocks to drive circadian rhythms 68. On the other hand, light's modulation of emotions does not rely on the SCN pathway but rather on the previously underappreciated ipRGCs- perihabenular nucleus (PHb) pathway. The PHb integrates with emotional regulation centers within a unique circuit, essential and sufficient for driving light's influence on affective behaviors 42. Their findings reveled that PHb neurons send collateral projections to ventromedial prefrontal cortex (vmPFC), dorsal and ventral striatum. The vmPFC is a cornerstone of mood regulation and has been persistently associated with major depressive disorders (MDD) through imaging studies of patients and the utilization of animal models of mood disorders 69,70. Among the PHb's projections, the dorsomedial striatum is a key target that is enmeshed within a thalamo-frontocortical circuit, potentially playing a role in the processing of affective-emotional information. In patients with MDD, a reduction in the volume of the caudate and putamen has been documented 71. Additionally, the ventral striatum, and more specifically the nucleus accumbens (NAc), represents another significant target of PHb neurons. The NAc has been extensively implicated in the modulation of mood and depression 72. In aggregate, their findings underscore the significance of the PHb as a hitherto underappreciated thalamic nucleus that is pivotal for mediating the impact of light exposure on mood states. In addition, recent research has illuminated a novel pathway through where light can modulate emotions by influencing the vmPFC via ipRGCs. Their findings suggest that light profoundly impacts the neuronal morphology, gene expression, network activity, and behavior of the mouse vmPFC through ipRGCs. Mice with ablated ipRGCs displayed impaired regulation of aversive emotions, a consequence attributed to the damage in the vmPFC 73. These results provide fresh insights into the neural basis required for light to influence emotions and learning.

The aforementioned experimental results aptly explain the effects of light on cognition and emotions. Irregular light stimuli can lead to PHb-related emotional changes, potentially due to increased PHb neuronal activity, sustained induction of the immediate early gene c-Fos by light, and disruptions in clock gene rhythmicity 42. These findings in mice may suggest that humans exposed to irregular light stimuli at night could experience similar neuronal changes, negatively impacting emotions and learning. Moreover, exposure to blue light-emitting LEDs has been demonstrated to decrease the expression of melanopsin and impair ipRGCs 74. In animal models, exposure to blue light LEDs resulted in mitochondrial damage, reduced dendritic arborization of ipRGCs, increased retinal GFAP immunoreactivity, and apoptosis in the outer nuclear layer of the retina 75. Over the past few decades, a significant trend has been the proliferation of artificial blue light sources and a marked increase in human exposure to blue light at night. The excessive exposure to blue light disrupts physiological ipRGC signaling, and aberrant ipRGC signals will lead to impairment of the vmPFC, which has been implicated in numerous psychiatric disorders. Numerous studies have reported cognitive declines in patients with severe depression, possibly due to neuroanatomical changes in these individuals 76,77. Therefore, a deeper understanding of the neural circuitry underlying emotions and learning, as well as the modulation patterns of light, holds promise for developing new treatments for neuropsychiatric disorders.

Light regulates emotions through various retinal-brain pathways. Anxiety is an adaptive response present in many species, enhancing alertness and vigilance against potential threats 78. Increasing evidence suggests that anxiety levels can be significantly modulated by lighting conditions. ipRGCs can also mediate prolonged anxiogenic behaviors induced by short-term acute exposure to intense light. Mice treated with a short (25-minute) acute exposure to intense light exhibited anxiety-like behaviors, observable for at least 20 minutes after exposure cessation. Further experiments indicate that this anxiety-like behavior is driven by increased melanopsin-driven ipRGC input to the central amygdala (CeA) and associated with upregulated corticosterone system activity. This response enables animals to maintain alertness for an extended period when encountering potential threats associated with high environmental lighting levels, thereby minimizing risk and aiding survival 79.

However, some questions remain unresolved, such as which subtype of ipRGC mediates the anxiolytic effect? It is reasonable to speculate that the M1 subtype of ipRGCs is key, as studies have found that anxiety-like behaviors disappear following selective ablation of ipRGCs using the immunotoxin melanopsin-saporin (SAP), which primarily targets M1 to M3 type ipRGCs 18,50,79. Given that the loss of melanopsin alone can eliminate the anxiolytic effect of light, and studies suggest that M1 drives melanopsin phototransduction while M2 and M3's light responses are mainly mediated by rod/cone input, there is reason to believe that the M1 subtype is the key ipRGC subtype exerting the anxiolytic effect 80. Future work using genetic mouse models with specific subtypes of ipRGCs eliminated or activated will provide strong clues for ultimately resolving this issue. In addition, the roles of other brain regions in the anxiolytic effect should not be overlooked. Besides the CeA, other brain areas such as the SCN, bed nucleus of the stria terminalis, and ventral geniculate nucleus (vLGN) that receive ipRGC projections play a role in modulating anxiety emotions 30,81. There are also complex interactions between the CeA and other brain regions 78,81. How these brain areas participate in the phenomenon of light-induced anxiety and their interactions await further exploration and validation through more sophisticated experiments in future research.

### 2.4. Brain development

In the intricate network of bodily connections, the relationship between the eyes and the brain is undeniably the most intimate. This connection allows for direct information exchange via the optic nerve, theoretically making the eyes the most influential organ on the brain. An increasing body of evidence suggests a close communication and mutual influence between the eyes and the brain.

Visual perception in mammals originates from the retina. During development, ipRGCs become photosensitive cells earlier than rods and cones 82. Recent research has discovered that light sensation mediated by ipRGCs promotes the synapse formation of various cortical and hippocampal pyramidal neurons. This phenomenon relies on the activation of ipRGCs and is mediated by the release of oxytocin into the cerebrospinal fluid by the SON and paraventricular nucleus (PVN). Further experiments have confirmed the direct connection between ipRGCs and oxytocin neurons in the SON, as well as the mutual projections between oxytocin neurons in the SON and PVN. Moreover, the lack of early cortical synapse formation promoted by light and mediated by ipRGCs impairs the learning ability of adult mice 1.

These findings demonstrate that early-life light enhances the level of oxytocin in the cerebrospinal fluid to promote cortical synapse formation through the ipRGCs-SON-PVN oxytocin neuronal circuit. This research underscores the importance of early-life light sensation for the development of learning abilities and its long-term impact on adult learning capabilities. Therefore, it is crucial for infants to be exposed to an appropriate light environment to foster their development.

### 2.5. Eye development

Sensory experience is pivotal in the development and maturation of the visual system 83. Traditionally, visual perception is mediated to the visual system via photoreceptors—rods and cones—which enable the system to utilize environmental cues for its development 84. However, the discovery of ipRGCs has illuminated the substantial impact of non-image-forming sensory experiences on visual system development and beyond 85. Indeed, ipRGCs mature prior to conventional photoreceptors and conscious vision, exerting a modulatory influence on retinal development. For example, the light-dependent vascularization of the retina and the tunica vasculosa lentis during the pre-eye-opening stage is melanopsin-dependent, with these RGCs playing an indirect role in sculpting the intraocular vascular network through their interaction with the immature vasculature. Beyond vascular development, ipRGCs are implicated in the regulation of retinal neuron number 86, the stratification of the ganglion cell layer 87, the development of the retinal clock 88, and the maturation of retinal waves 89. These findings suggest that light perception plays a fundamental and extensive role in the development of the retinal landscape before eye opening. Collectively, these findings underscore the fundamental role of light perception in shaping the retinal landscape antecedent to eye opening.

In a recent study, the role of photoreceptors in ocular development has been further elucidated, revealing that ipRGCs facilitate the apoptosis of Nrl-positive rod precursors localized to the inner nuclear layer and inner neuroblastic layer 90. This process is light-dependent, with ipRGCs releasing glutamate that is detected by the transiently expressed glutamate receptor Grik3 on rod precursors. These signaling molecules are capable of promoting rod precursor cell death and modulating their numbers. Furthermore, through histological and informatic analysis of human fetal retinas during development, it has been observed that the fundamental characteristics of this developmental pathway are conserved. These findings offer new insights into the understanding of retinal development and adaptive mechanisms, potentially informing therapeutic strategies for retinal diseases. Additionally, the study suggests that the regulation of the light-eye-body axis is not limited to neural circuits; photoreceptors may also directly secrete neurotransmitters after receiving light signals, regulating downstream processes through paracrine and immune pathways. This aspect of research has not been previously emphasized, and future studies could explore other regulatory pathways of the light-eye-body axis, such as paracrine and immune mechanisms.

### 2.6. Gut microbiota

The gut microbiota is widely regarded as a vital, dynamic ecosystem within the human body, playing a significant role in both health and disease 91. The composition and relative abundance of the gut microbiota are influenced by various factors, including genetics and diet. Even under normal conditions, daily fluctuations in the relative abundance of many types of gut microbiota are observed 92. While the physiological significance of these daily changes remains unclear, their role in disease states is becoming more evident. Studies have found that dysbiosis of the gut microbiota can predict the risk of type 2 diabetes, and in animal models of Parkinson's disease, an imbalance in the gut microbiota precedes the onset of neuropathological symptoms 93,94.

Recent research has revealed that external light-dark cycles can influence the gut microbiota through ipRGCs. The normal alternation of light and dark is one of the primary drivers of the daily variations in gut microbes, as the percentage of oscillating gut microbiota significantly decreases under constant darkness or dim light at night. In this process, ipRGCs are crucial for the daily fluctuations of the gut microbiota driven by signals from melanopsin and rod and cone photoreceptor cells. Compared to control mice, melanopsin gene knockout mice exhibit a different diurnal pattern of gut microbiota, although the overall percentage of change is similar. This may be due to the partial compensation of rod and/or cone cell signals for the loss of melanopsin signals. In mice with ablated ipRGCs, the daily fluctuations of the gut microbiota are nearly absent. Unlike the daily variations, the composition of the gut microbiota is directly regulated by ipRGCs and melanopsin. The composition of the gut microbiota changes significantly under LD and dLAN conditions, and both melanopsin gene knockout and ipRGCs ablation can counteract these changes. Thus, activating melanopsin and/or ipRGCs-mediated light information can influence the composition and diversity of the gut microbiome. Interestingly, ipRGCs drive the light-induced daily changes in gut microbiota through the sympathetic nervous system, while the composition of the gut microbiota is affected by an ipRGCs sympathetic-independent unknown pathway 95.

In summary, ipRGCs are an important mediator for the regulation of the gut microbiota by light exposure through the eyes. External light exposure can modulate the composition and daily variations of the gut microbiome via the melanopsin signal-ipRGCs pathway, highlighting a novel aspect of the intricate interplay between our environment and internal physiology.

### 2.7. Hair generation

Human hair forms a protective barrier against external harm, assists in maintaining body temperature, and importantly, scalp hair serves a significant aesthetic function, enhancing feelings of happiness and social acceptance 96. Human hair originates from hair follicles (HF), which are complex, tiny organs abundantly present in the dermis and subcutaneous tissue. They undergo repeated regeneration cycles, including growth, regression, and rest phases, making them one of the few organs that undergo cyclical degeneration and regeneration throughout life 97. The growth of hair follicles is primarily driven by hair follicle stem cells (HFSC), thus the regulation of HFSC activity becomes key to hair growth 98. HFSC activity has been shown to be regulated by intrinsic cellular signaling pathways as well as signals from the local and systemic environment 99,100.

As one of the many regulators of HFSC, light can stimulate hair growth directly through skin irradiation and indirectly through the eyes 97. In mice, short-wave blue light stimulation to the eyes can significantly induce hair regeneration. In this process, although rods and cones play a certain role, the projection of M1 ipRGCs to the brain's SCN is necessary. Upon receiving external light stimulation, the human body can activate systemic sympathetic activity in the skin to increase the release of norepinephrine to promote HF regeneration through the ipRGCs-suprachiasmatic nucleus-sympathetic nerve circuit. For HFSC cells themselves, upon receiving external light stimulation, the Hedgehog signaling pathway is activated and is necessary for eye light-induced HFSC activation and the initiation of the growth phase 101.

The discovery of the indirect regulatory mechanism of hair follicle growth by light through the eyes is very meaningful. Although both are projected by ipRGCs to the SCN, unlike the light synchronization of circadian rhythms, this pathway changes peripheral SC activity by activating the sympathetic nervous system and has an immediate effect, which can respond immediately to changes in external light. This also shows the multifunctionality of the ipRGCs-SCN pathway in regulating long-term daily oscillating activities and acute light responses. This also extends another question, since both circadian rhythm light induction and acute light activation of the sympathetic nerves are mediated by the ipRGCs-SCN pathway, is it because the functions of the two pathways are dominated by different subgroups of ipRGCs? Answering this question can be further improved by single-cell sequencing technology and in situ targeted gene knockout technology.

This discovery greatly expands the range of human body functions affected by light through the eyes. And in this, there are two questions worth thinking about: In addition to HFSCs, SCs in other tissues are also regulated by the autonomic nerves 102. So, are SCs in other tissues affected by the ipRGCs-SCN-sympathetic nerve axis? And because light-activated sympathetic nerves are not limited to the skin, this pathway may also play a more widespread role in coordinating systemic functional responses that have not been discovered, which is also worth studying 103.

### 2.8. Pigmentation

Skin pigmentation, modulated by light, is a phenomenon that occurs in virtually all organisms. In invertebrates or lower vertebrates, there are two mechanisms of light-regulated skin pigmentation: physiological pigmentation and morphological pigmentation. Physiological pigmentation occurs relatively quickly, involving changes in the distribution of pigment granules in the cytoplasm of pigment cells, which is significant for diurnal rhythm changes or camouflage 104. In contrast, morphological pigmentation occurs more slowly, requiring changes in the number or type of pigment cells, typically occurring with seasonal changes 105.

For physiological pigmentation, it is often assumed that it originates from the direct response of melanin to light, as studies have shown that light signals can indeed be recognized by cells and converted into intracellular signals, triggering the movement of melanin groups leading to pigmentation 106. However, there is also a “secondary color response” in light-regulated physiological pigmentation. Using the african clawed frog as a good model for melanopsin research, researchers found that light activation of melanopsin in ipRGCs inhibited the expression of pro-opiomelanocortin and alpha-melanocyte-stimulating hormone (α-MSH) synthesis in the intermediate layer of the pituitary, both of which can promote pigmentation 107. As for morphological pigmentation, although light can also regulate morphological pigmentation through ipRGCs, it seems that different circuits and molecular mechanisms control physiological and morphological skin pigmentation separately, and the visual pathway of morphological pigmentation has not been clearly elucidated 108.

In summary, the eye controls skin darkening through two different pigmentation mechanisms, both of which involve the regulation of inhibitory synapses, as reduced eye activity will cause a certain pigmentation response. Although the retinal circuit controlling morphological skin pigmentation is not clear, it is interesting that light-induced physiological pigmentation always occurs simultaneously with morphological pigmentation, suggesting that there may be neuronal and/or endocrine connections between these two different circuits 108. However, it must be acknowledged that their research was conducted in African clawed frogs, and the neuroendocrine circuits in humans will be more complex, so further research is needed to determine whether this pathway is valid in humans.

### 2.9. Itch behavior

The survival of animals largely depends on obtaining information from the external environment through vision 109. In addition to direct observation, social animals can infer and adapt to the ever-changing living environment by observing and imitating the behavior of conspecifics, which can predict potential dangers that are sometimes difficult to detect 110. Contagious itch behavior (CIB) is one of them, which is an involuntary imitation behavior formed during evolution, different from the social imitation behavior acquired by learning 111. Previous research has shown that during the propagation of itching behavior in mice, the neuronal activity in the SCN of the hypothalamus increases. The ablation of gastrin-releasing peptide receptor (GRPR) or GRPR neurons in the SCN eliminates contagious scratching behavior, and the activation of SCN GRP/GRPR neurons induces scratching behavior, indicating that GRP-GRPR signal transduction is necessary and sufficient for transmitting contagious itch information in the SCN 112. Their research provides a molecular and neural basis for contagious itch behavior in mice. Based on their research, Gao and others have wonderfully extended this pathway to both ends. Upstream of the pathway, they proved that it is ipRGCs that play a role in conveying contagious itch information. Downstream of the pathway, they found that GRPR neurons project to paraventricular nucleus of the thalamus (PVT) to transmit itch information. This indicates that the retina-ipRGCs-SCN-PVT pathway is a non-classical subcortical visual pathway, which is necessary and sufficient for mediating CIB in a melanopsin-independent manner 113.

Their research reveals an unexpected function of ipRGCs in mediating motion-based visual stimuli, similar to classical RGCs, thereby significantly expanding the function library of ipRGCs, from its classical melanopsin-mediated non-image-forming function to melanopsin-independent image-forming function. At the same time, their research may help us understand the origin of emotional contagion. CIB is easily regarded as an instinctive action imitation behavior, which is ubiquitous in lower vertebrates and invertebrates 114. At the same time, CIB is not an emotional contagion, which is limited to familiar individuals in mice 115. Visually induced scratching actions can be simulated by artificially stimulating GRPR neurons in SCN, indicating that the cascading neural circuit of itching is automatically activated in this process, rather than the manifestation of more cognitive/emotional abilities required for emotional rendering 116. Therefore, it is interesting to further study whether the ipRGCs-SCN circuit can mediate other types of social contagious behaviors, such as yawning.

### 2.10. Body temperature

A wealth of evidence, including animal and clinical experiments, suggests that light can significantly influence body temperature 117,118. Notably, changes in body temperature are most sensitive to stimuli from short-wavelength light 119. Existing research indicates that melanopsin is most sensitive to light at 480nm, leading us to hypothesize that the regulation of body temperature by light is mediated by ipRGCs 120. Building on this, Rupp and his colleagues discovered that the regulation of body temperature and sleep by light share similar characteristics, both involving indirect regulation through circadian rhythm light synchronization and direct acute regulation 44. Their results suggest that ipRGCs are the only retinal cells that acutely regulate body temperature in response to light. Interestingly, Brn3b (-) and Brn3b(+) ipRGCs play distinctly different roles in this process. Brn3b(-) ipRGCs project to the SCN to mediate the circadian rhythm light synchronization of body temperature, while Brn3b(+) ipRGCs project to other parts of the brain and are necessary for the acute regulation of body temperature. Their results further indicate that Brn3b(+) ipRGCs mediate the acute effect of light on body temperature through projections outside the SCN, while Brn3b(-) ipRGCs mediate the circadian rhythm light entrainment of body temperature through projections to the SCN and/or intergeniculate leaflet (IGL).

The significance of this research lies in demonstrating that the connection of ipRGCs to the SCN to control circadian rhythm light synchronized electrical activity does not control the direct effect of light on body temperature. Instead, separate circuit extending from ipRGCs to unknown parts of the brain directly influences changes in body temperature. Body temperature plays a crucial role in cognition and alertness 121. A better understanding of this regulatory circuit could enable researchers to develop methods to help emergency personnel or night shift workers stay awake and alert at night, while minimizing disruption to circadian rhythms.

### 2.11. Memory

As previously stated, changes in light exposure can have profound effects on human physiological functions. Memory, as one of the core components of cognitive neurobehavioral performance, is also regulated by light. While it is obvious that image-forming vision is central to memory formation, recent research has found that non-image-forming vision can also regulate various types of memory. Memory can be broadly divided into declarative memory (the ability to recall events or facts) and non-declarative/procedural memory (the ability to execute learned skills or activities) 122. In rodents, bright light has been shown to enhance fear and spatial memory 123,124. Animals raised under dim and irregular light conditions show impaired spatial memory 124. There is a correlation between light intensity and the downregulation of object and odor recognition memory 125. This section will discuss how light regulates the formation of various types of memory through the eyes.

In cognitive psychology and neuroscience, spatial memory is a form of memory responsible for recording and recalling the information needed to go to a certain location, and recording the location of objects or the location of events. There have been many studies on the impact of light on spatial memory. However, research on the neural mechanisms of light affecting cognitive functions and the role of the eye in this process is relatively scarce. Research by Huang Xiaodan and others found that long-term bright light therapy can promote spatial memory through a binocular visual circuit related to the reunion nucleus (Re) 41. Specifically, they proved that a subgroup of ON-type retinal ganglion cells expressing SMI-32 can innervate CaMKIIα neurons in the vLGN and IGL of the thalamus, and then activate the reunion nucleus Re to promote spatial memory. And separately activating a part of the RGCs subgroup projecting to vLGN/IGL, a part of the vLGN/IGL neurons projecting to Re, and Re neurons are all effective. In summary, this study found a new memory-related light information transmission pathway, and the authors proved that phototherapy information can enhance spatial memory ability through the mediation of the retina-vLGN/IGL-Re pathway 41.

Social recognition memory is crucial for the survival of social species and is necessary for group life, selective reproduction, pairing and dominance hierarchy 126. Recent research results show that acute light exposure can impair social recognition memory in mice. Specifically, bright light can transmit signals to pSON through M1-type ipRGCs and activate GABA neurons in it, and inhibit oxytocin neurons in SON 127. Oxytocin plays a key role in social recognition memory, existing research shows that male mice lacking oxytocin show a decline in the ability to recognize female mice 128. Interestingly, the above conclusions were obtained in the morning experiment, but when the same experiment was repeated at noon, whether or not bright light was given, the mouse's social recognition memory was close to zero. The difference between the morning and noon experimental results suggests that the excitability of SON neurons and the release of oxytocin have circadian fluctuations that may have some impact on the results 129. Further research is needed to explore the specific mechanism of SRM reduction at noon.

A recent study explored how light affects learned fear in mice. They used a mature measurement method to assess memory ability: voice-induced fear conditioning. When a tone is repeatedly presented and accompanied by a slight electric shock, the subject will associate this tone with the electric shock, and even if only the tone is heard without the electric shock, a fear response will occur 130. In rodents, the response to fear is freezing (completely stopping activities). This indicator is stable and measurable, and can be used as an indicator of mouse memory ability 130. When the researchers used light with a wavelength of 470nm to stimulate mice lacking rod cells and cone cells or melanopsin knockout mice, the results showed that in the control group and melanopsin gene knockout mice, the light applied during the acquisition and recall process enhanced the freezing response, which is a manifestation of the enhanced learning and memory ability of the test mice. But there is no such effect in mice lacking rod cells and cone cells, indicating that this effect is driven by rod cells and cone cells 123. In a mouse experimental study, researchers found that object recognition memory does not require visual input, because even in the absence of rod cells and cone cells, mice can still successfully distinguish between novel and familiar objects. But visual spatial memory (that is, memory of where the object is) requires classical photoreceptors. At the same time, their results show that under strong light, the mouse's object recognition memory will be disturbed, but this effect is completely eliminated in mice lacking melanopsin, indicating that the effect of strong light on recognition memory performance is mediated by ipRGCs and rods and cones.

The above research results fully demonstrate the impact of light on memory through the eyes. However, memory is extremely complex, whether it is declarative memory or non-declarative memory, it involves a series of complex processes, including acquisition, encoding, consolidation, maintenance and retrieval, and they can be subdivided into multiple types 131. Current research only confirms that some types of memory can be regulated by light signals received and transmitted by the retina, but in fact the impact of light signals on various types of memory has been widely studied. Blue light can improve language memory performance during memory consolidation, sunlight can increase the synaptic release of glutamate and enhance the motor learning and memory ability of mice, the retinal-neural mechanism behind these phenomena is worth exploring 132,133. The functions of ipRGCs and many brain areas are highly conserved between mice and humans, so mice can be used as a suitable animal model to explore the neural circuit of light affecting memory 134. The results obtained from mouse experiments can also deepen our understanding of the impact of light on various memory mechanisms.

## 3. The role of light signals through the eye in regulating disease throughout the body

**Figure 3:**
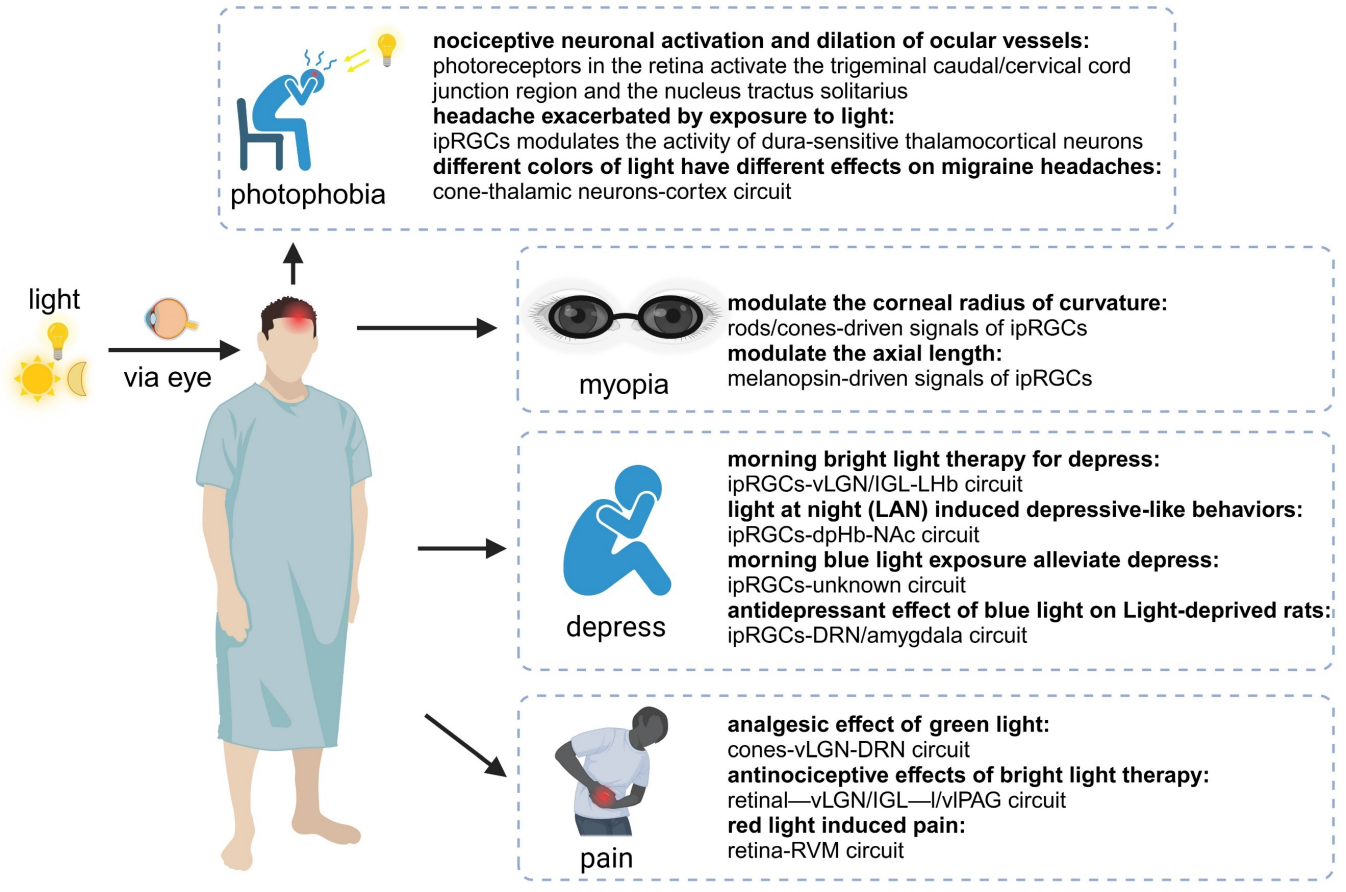
** The light-eye-body axis has a pervasive influence on the organism's pathological states.** The light-eye-body axis affects pathological states through a wide range of pathways. IpRGCs: intrinsically photosensitive Retinal Ganglion Cells; vLGN: ventral geniculate nucleus; IGL: intergeniculate leaflet; LHb: lateral habenula; dpHb: dorsal perihabenular nucleus; NAc: nucleus accumbens; DRN: dorsal raphe nucleus; IGL: intergeniculate leaflet; l/vlPAG: lateral/ventral lateral parts of the periaqueductal gray area.

Beyond the role in regulating physiological functions, recent studies increasingly revealed that the light-eye-body axis also plays a crucial regulatory role in various pathological processes such as myopia, photophobia, and depression (Figure 3).

### 3.1. Photophobia

Photophobia is a sensory disturbance caused by light, mainly manifested by migraine. The term originates from two Greek words: "photo" meaning "light" and "phobia" meaning "fear," together signifying a "fear of light." Patients may experience photophobia due to various diseases, including primary ocular conditions, central nervous system disorders, and psychiatric illnesses 135. Light, being the primary stimulus for photophobia, implicates the involvement of photoreceptors and the light perception pathway in the pathophysiology of photophobia. A clinical trial has revealed that intense light stimulation can diminish the pain perception thresholds of the trigeminal and cervical nerves through the visual system, thereby precipitating migraines. This finding suggests that the visual pathway plays a significant role in modulating pain signals associated with migraines, offering a novel perspective on the interplay between light exposure and headache disorders 136. Recent evidence increasingly suggests that melanopsin signaling systems can mediate photophobia, and ipRGCs play a significant role in the pathophysiology of photophobia, independent of rods and cones 137.

Among these evidence, two distinct neural pathways have been clearly described, detailing how ipRGCs transmit noxious light signals to the brain. The team led by Okamoto explored the first pathway, focusing on the role of the ophthalmic branch of the trigeminal nerve in photophobia. They employed quantitative Fos-like immunoreactivity to determine the pattern of neuronal activity in the caudal brainstem of anesthetized rats following intense light stimulation of the retina. This demonstrated that photoreceptors in the retina activate the trigeminal caudal/cervical cord junction region and the nucleus tractus solitarius, ultimately leading to nociceptive neuronal activation and dilation of ocular vessels 138. However, their research did not extend to the mechanisms of retinal light signal transduction in migraines. Another study described the second pathway of photophobia, utilizing single-unit recording and neural tract tracing in rats to identify the axons of dura-sensitive neurons in the posterior thalamus, which project extensively to layers I-V of the somatosensory, visual, and associative cortices, with their activity being significantly modulated by light. The perception of migraines is mediated by noxious signals transmitted from the cranial dura mater to the brain. Intriguingly, they discovered that these dura-sensitive neurons in the posterior thalamus are connected to the axons originating from ipRGCs in the retina. They proposed that photoregulation of migraine headache is exerted by ipRGCs-dura-sensitive thalamocortical neurons 137. Using diffusion MR tractography, Maleki and colleagues also found similar conclusions in human 139.

The aforementioned studies firstly reveal the retinal mechanisms of photophobia: it is mediated by ipRGCs and influences photophobic behavior by modulating the activity of dura-sensitive thalamocortical neurons 137. This also explains why patients who retain non-image-forming vision yet are blind still exhibit symptoms of photophobia, while those who have lost the optic nerve or eyes do not 139.

Previous research has highlighted the integral role of ipRGCs in the pathogenesis of photophobia migraine, with ipRGCs exhibiting heightened sensitivity to blue light. Clinical evidence has consistently pointed towards the exacerbating effects of short-wavelength (blue) light on migraines, and the mitigation of symptoms through the use of devices that block blue light, such as sunglasses, has reinforced the notion that blue light might be the primary cause of photophobia migraine 140. However, this concept has been derived from blind migraine patients lacking cone and rod photoreceptors, potentially overlooking the influence of other photoreceptive cells 141 In fact, the impact of different colors of light on migraines can be varied and even contradictory. Psychophysical assessments conducted on patients with normal vision have revealed that the likelihood of green light exacerbating migraines is significantly lower than that of white, blue, amber, or red light. Through electroretinography and visual evoked potential recording in patients, and multi-unit recording of dura- and light-sensitive thalamic neurons in rats, it has been discovered that the photophobic response to colors and migraines may originate from cone-driven retinal pathways and be relayed through light-sensitive trigeminovascular thalamic neurons to the cortex. This finding elucidates why green light has the weakest migraine "effect," as it activates the cone-mediated retinal pathways the least. Green, white, and blue lights can induce migraines through this pathway, while red light, which does not activate thalamic neurons, and amber light, which has not been further investigated by the group, may trigger migraines through cone-unknown pathways. This study underscores the differential effects of various colors of light on migraines, with a particular focus on the pivotal role of cone cells 142. Green light is the least likely to aggravate migraines and, at low intensities, may even serve a therapeutic role by reducing headache intensity. The soothing effects of green light may involve complex psychobiological mechanisms 143. Indeed, green light possesses inherent analgesic properties, the mechanisms of which will be elaborated in subsequent sections.

### 3.2. Depression

Phototherapy, a non-pharmacological intervention based on circadian rhythms, has been proven effective and safe for various depressive disorders, yet the underlying mechanisms continue to perplex researchers globally 144,145. Conversely, studies have reported that nocturnal light exposure may increase the risk of depressive symptoms 146,147. The contradictory effects of light exposure across various circadian phases suggest that the timing of light exposure may be a critical factor1.

Morning bright light therapy (BLT) has been established as a safe and effective treatment for depression 144. Phototherapy exerts its antidepressant effects through photosensitive neural circuits, with the retinal-vLGN/IGL-LHb pathway being one of the neural routes for BLT's antidepressant action. Specifically, BLT activates GABAergic neurons in the vLGN/IGL governed by M4-type ipRGCs, then inhibiting aberrant firing of CaMKIIα neurons in the LHb, thereby alleviating depressive-like behaviors in mice 148. In addition, morning blue light exposure can significantly alleviate depressive symptoms through ipRGCs, exhibiting a profound antidepressant effect 149. Extended exposure to blue light for 12 hours during the day has been demonstrated to possess a pronounced antidepressant effect on the depressive phenotype in rats subjected to light deprivation. This therapeutic effect may be attributed to activating the serotonergic system of DRN and brain-derived neurotrophic factor signaling pathway in the amygdala 150. The impact of phototherapy on hormones may also contribute to its antidepressant mechanism. Melatonin, a hormone that regulates circadian rhythms and promotes sleep, produced and secreted by the pineal gland, is modulated by phototherapy. Activation of ipRGCs inhibits pineal function, reducing melatonin synthesis and release during the day, thus restoring the biological clock to homeostasis 151.

However, it is crucial to emphasize that while morning bright light can improve depressive symptoms, nocturnal light exposure can have adverse effects. Kai An's team found that light at night (LAN) induced depressive-like behaviors in mice without disturbing circadian rhythms. This effect was mediated by a neural pathway from ipRGC to the dorsal perihabenular nucleus (dpHb) and then to the NAc. Notably, the dpHb, controlled by circadian rhythms, is more excitable at night than during the day. This suggests that the ipRGCs→dpHb→NAc pathway preferentially transmits light signals at night, mediating LAN-induced depressive-like behaviors 152.

Phototherapy is a low-cost and effective treatment modality for various diseases. Numerous studies have validated its efficacy for patients with different types of depression, yet many mysteries remain regarding the mechanisms by which phototherapy produces its effects 145. The LHb, a highly conserved part of the epithalamus across species, is implicated in the increase of depressive-like behaviors when activated, while its inhibition improves depressive symptoms 153. Huang and colleagues revealed that the mechanism of phototherapy involves intense light suppressing LHb activity via the M4-type ipRGCs directly governing the vLGN/IGL-LHb pathway, exerting an antidepressant effect 152. The increased risk of depression associated with nocturnal light exposure is due to the dpHb within the ipRGCs→dpHb→NAc pathway being more excitable at night, thus preferentially responding to LAN rather than daylight, regulating NAc activity, a brain region highly associated with depression, and consequently inducing depressive-like behaviors. Therefore, the increased risk of depression when light occurs during the “wrong” phase of the circadian cycle is well explained.

Nighttime light pollution has become increasingly severe, with over 80% of the global population suffering from significant nocturnal light pollution 154. Beyond lighting devices, the proliferation of smart devices such as smartphones, tablets, and computers has also become a source of LAN. In An and colleagues' experiments, the light source used to stimulate ipRGCs and induce depression was environmental blue light at 473nm, indirectly validating the value of electronic device eye protection modes (reducing screen blue light). Future research could explore the effects of light of different wavelengths on depression. In this industrial age, we must be vigilant about the harm caused by nighttime light pollution.

Many mood disorders, including Seasonal Affective Disorder, Major Depressive Disorder (MDD), and bipolar disorder, are intimately linked to abnormal sleep and circadian rhythms 145. Environmental disruptions to normal sleep/wake patterns, light-dark changes, and seasonal variations can trigger these disorders, while sleep/wake pattern abnormalities are also one of the clinical diagnostic criteria for mood disorders. Evidence suggests that treatments targeting circadian rhythms (such as acute sleep deprivation, BLT, and phase advance of sleep) can successfully treat depression 155. Clinical evidence indicates that morning BLT, alone or in combination with fluoxetine for 8 weeks, is well-tolerated and effectively reduces depressive symptoms in patients with MDD, as demonstrated by changes in the Montgomery-Asberg Depression Rating Scale scores 155. One potential mechanism for BLT's antidepressant effect on MDD is the ipRGCs-vLGN/IGL-LHb pathway proposed by Huang and others. From our previous discussion, we know that the ipRGCs-SCN axis is crucial for controlling the biological clock. Thus, leveraging the key node of ipRGCs in phototherapy to cure depression while also positively influencing the biological clock may be a direction for future consideration in phototherapy.

### 3.3. Pain

Light therapy has been reported to have unexpected effects on various diseases. It is widely used clinically for a range of conditions, including depression, dementia, and circadian rhythm disorders 156-158. Regarding pain, phototherapy has been shown to produce different effects depending on the intensity, wavelength, and pathway of the light exposure. For instance, green light exposure has been proven effective in alleviating pain in conditions such as migraines and fibromyalgia 159,160. Bright light can treat chronic back pain, while low-intensity light can relieve pain caused by a range of musculoskeletal diseases and neuropathic pain resulting from sciatic nerve damage 161-163. Another study found that white, blue, amber, and red light exacerbated migraines to a greater extent in patients with episodic migraines 164. Clearly, light plays a crucial role in inducing pain and analgesia, making it necessary to study its specific mechanisms and the role of the eye in this process.

Rajesh and colleagues have elucidated an injury-free model of functional pain in rats through exposure to red light 165. In their study, rats were subjected to red light-emitting diodes (RLEDs) with an intensity of 50 lux, for 8 hours daily over a period of 5 days, which resulted in thermal hyperalgesia and mechanical allodynia. The intriguing aspect of this research is that when rats were equipped with contact lenses used to block light, the aforementioned effects were abolished, thereby highlighting the indispensable role of the visual system in mediating such nociceptive responses. Furthermore, their findings underscore the necessity of activation of downstream pathway in the rostral ventromedial medulla (RVM) in sustaining the thermal hyperalgesia and mechanical allodynia induced by RLED exposure. It is well-documented that neurons within the RVM project to the spinal or medullary dorsal horns, where they can either directly or indirectly modulate nociceptive transmission 166. By microinjection of bicuculline, a GABA-A receptor antagonist, to suppress the activity of the RVM, the thermal hyperalgesia and mechanical allodynia effects induced by RLED were mitigated. This suggests that the modulation of RVM activity is a pivotal factor in the manifestation of pain behaviors elicited by red light exposure 165. While a plethora of studies have suggested analgesic effects of red light, the aforementioned research presents a contrasting viewpoint. This discrepancy may arise from the longer wavelength of red light, allowing it to penetrate the skin and exert its analgesic effects independently of the visual system, directly acting upon peripheral skin tissues 167. Given the dualistic effects of red light, with central mechanisms exacerbating pain and peripheral mechanisms alleviating it, it is prudent to consider minimizing intraocular exposure to red light in the formulation of future phototherapy standards. This approach will ensure a balanced therapeutic intervention that maximizes the beneficial analgesic effects while mitigating any potential adverse effects on pain perception.

Studies have confirmed that the eyes indeed participate in the analgesic effect of green light, as the original analgesic effect of green light disappeared after mice were fitted with opaque glasses 168. Another study showed that green light-emitting diodes alleviate neuropathic pain by activating glutamatergic neurons in the vLGN, while RLEDs promote pain using GABAergic neurons in the vLGN. However, the study did not investigate the role of the retina in this process 169. The research that truly demonstrates the bridging role of the eye in light-mediated pain/analgesia comes from Professor Hu's team. They proved that under strong light exposure, RGCs in the mouse retina directly project to GABAergic neurons in the vLGN/IGL, which in turn directly project to inhibit the activity of GABAergic neurons in the lateral and ventral lateral parts of the periaqueductal gray area (l/vlPAG), thereby regulating pain-related brain regions such as the locus coeruleus (LC) and the RVM. Specific activation of the vLGN/IGL—l/vlPAG pathway can increase the pain threshold in wild-type mice and significantly improve pain symptoms in mice with inflammatory and pathological pain models. This study elucidates the key role of the retinal—vLGN/IGL—l/vlPAG pathway in mediating strong light analgesia 170. Interestingly, a recent study found that cone cells can also independently dominate the analgesic effect of green light. In a mouse arthritis model, the analgesic effect of green light was not affected by the ablation of ipRGCs, while the ablation of cone cell photoreceptors completely inhibited the analgesic effect and rod ablation only partially reduced pain relief. Their findings indicate that cone-dominated retinal inputs mediated green light analgesia through the vLGN-DRN pathway and suggest that this signaling pathway could explain the analgesia effect of green light therapy in clinic 171.

Bright light therapy can have beneficial effects on various human functions, including pain, mood, and cognition 172,173. In this section, we discuss the mechanisms of bright light's analgesic effects based on existing research evidence. Combining the description of the positive effects of bright light on depression in the previous section, we can see that they share a similar mechanism. Both are achieved through the RGCs-vLGN/IGL pathway, with the difference being the activation of different downstream brain regions. These conclusions may indicate that bright light can activate some protective mechanisms in the human body, with the core pathway being the RGCs-vLGN/IGL pathway. Future research can focus on the relationship between other effects of bright light therapy and this pathway.

The analgesic effects of different colors of light, including green, blue, red, and near-infrared, have been proven by research 167,174. Longer-wavelength light (such as red light: 625-740nm) has high tissue penetration and can provide analgesia through skin irradiation 175. Green light, with its shorter wavelength, does not seem to provide systemic analgesic effects through the skin. In a mouse experiment, researchers found that the analgesic effect of green light required the involvement of the visual system 168. All the above evidence indicates that the analgesic mechanism of green light differs from that of other colors of light and may be mediated by the eyes. The study by Tang et.al. proved that the analgesic effect of green light depends on the rods-RGCs-vLGN-DRN pathway. The significance of this mechanism discovery lies in the identification of an analgesic pathway independent of ipRGCs, where green light is projected to the vLGN through rods via “conventional” (non-photosensitive) ganglion cells, and enkephalinergic neurons in the vLGN then project to the DRN to mediate green light. However, their conclusions do have limitations. The melanopsin-SAP they used preferentially ablates M1-M3 type ipRGCs, while M4 to M6 type ipRGCs are essentially not ablated 18,22. Previous research reported that ipRGCs responsible for strong light damage perception are mostly M4 type 176. Therefore, it is necessary to use more effective techniques to ablate all subtypes of ipRGCs to strengthen their conclusions. Green, as a natural color, can alleviate physiological and psychological pain 177. Psychological research suggests that “green” represents positive information related to happiness 178. Given the many benefits of green light to the human body, and the relative economy and safety of green light analgesia methods, it is necessary to deeply explore the potential mechanisms of green light analgesia.

### 3.4. Eye disease

#### 3.4.1. Retinal disease

Light, especially blue light, can cause damage to photoreceptors which is associated with some eye diseases 74. IpRGCs are reduced in number and impaired in function in patients with glaucoma and diabetic retinopathy 179-181. It is evident that blue light activates apoptosis in ipRGCs, which may contribute to the onset or exacerbation of glaucoma 182. Previous studies have reported a higher prevalence of anxiety, depression and sleep disturbances in patients with glaucoma, yet the precise mechanisms underlying this phenomenon remain elusive 183. Given the established association of ipRGCs with these functions, we hypothesize that the increased prevalence of sleep disorders, anxiety and depression in glaucoma patients may be attributed to glaucoma-induced damage to ipRGCs, resulting in dysregulation of the corresponding non-image-forming functionalities.

#### 3.4.2. Myopia

At the same time, light can also indirectly affect certain eye diseases through photoreceptors, such as myopia. Myopia is the most common ocular disorder worldwide, with its disease burden escalating. It is estimated that by 2050, nearly five billion individuals will be affected by myopia, with 900 million suffering from high myopia 184. Research into the mechanisms of myopia lays the foundation for establishing effective prevention and control strategies, addressing a significant need in human society. Among the myriad studies on myopia mechanisms, Professor Xiongli Yang's team has blazed a trail by exploring the role of ipRGCs in the onset and progression of myopia 36.

Myopia is a pathological refractive condition of the eye, caused by abnormal growth of ocular media and/or refractive power, resulting in the formation of a visual image in front of the retina, leading to blurred vision 185. The research by Professor Yang's team indicates that selective destruction or specific activation of ipRGCs in developing mice leads to significant myopic or hyperopic refractive shifts, respectively. Further experiments revealed that melanopsin signals within ipRGCs, along with rod/cone signals, respectively modulate ocular development by influencing axial length (AL) and corneal curvature radius (CRC). Given the contribution of ipRGCs to axial development, it is reasonable to predict that myopia may be regulated by signals mediated by ipRGCs. Notably, subsequent experiments found that in a form-deprivation myopia mouse model, the expression levels of melanopsin in the eye's ipRGCs and the amplitude of their light response were upregulated. Knocking out melanopsin or rearing mice in an environment devoid of 480 nm wavelength light (the peak excitation wavelength for melanopsin) resulted in downregulation of melanopsin activation, significantly reducing the effects of form-deprivation induced myopia, highlighting the substantial impact of ipRGCs on the development of myopia 185.

This work elucidates for the first time the pivotal role of ipRGCs in ocular development and the formation of myopia, offering novel insights for intervening in the onset and progression of myopia. This can be achieved by employing light with a carefully designed spectral composition that primarily activates rods and cones while avoiding melanopsin activation or using melanopsin inhibitors, thereby slowing the progression of myopia and/or reducing its severity 185. However, the study did not clarify how melanopsin and rod/cone signals (both propagated via ipRGCs) differentially affect AL and CRC. Two potential avenues for further investigation are the complex structural-functional interactions between ipRGCs and dopaminergic amacrine cells, as well as the profound interplay between ocular circadian rhythms and retinal vasculature in influencing ocular development/myopia 186,187.

## 4. Applications of light-eye-body axis for improving health

The conception of light-eye-body axis provided a comprehensive framework for understanding the intricate effects of light on human physiology, behavior, and pathogenesis. It unveiled the potential for light to serve as a non-invasive modulator of biological functions, thereby presenting novel avenues for enhancing life quality. It beckons a convergence of expertise across disciplines that have traditionally operated in silos: vision science, neuroscience, architectural lighting. The integration of these fields opens up a plethora of opportunities to harness light's therapeutic potential, with direct bearings on health and disease management. The light-eye-body axis thus stands at the forefront of a new interdisciplinary paradigm, promising significant advancements in how we approach human well-being and the treatment of various conditions.

### 4.1. Diagnostic procedures for the melanopsin system

Abundant research evidence suggests that disruptions in melanopsin signaling may underlie several human diseases, including sleep disorders, seasonal affective disorders, and depression 137. Reliable and straightforward methods to quantify melanopsin function are essential for determining whether the melanopsin system influences these pathological states. However, almost all current retinal diagnostic procedures measure the structure and function of retinal outer rod and cone photoreceptors, neglecting the assessment of ipRGC function. A promising approach is the analysis of pupillary light reflexes. Three types of photoreceptor cells contribute to the pupillary light reflexes, but melanopsin-dependent pupillary responses are sensitive to short-wavelength light and are slow and sustained. Studies have confirmed that a 10/20-second light stimulation protocol can relatively independently detect ipRGC function in humans and rhesus monkeys 188,189. In summary, the assessment of ipRGC function will be the starting point for further distinguishing between patients with complete loss of NIF and IF vision and those who have only lost IF vision. This classification will help determine whether normal ipRGC function in some blind patients is more beneficial for maintaining a better quality of life than in patients without light perception.

### 4.2. Pharmacological intervention

The unique properties of melanopsin and the rod/cone phototransduction pathways encouraged the idea of manipulating signal traffic via ipRGCs to manage human diseases that depend on lighting conditions. However, the focus of intervention remains unclear. The ideal strategy would be to modulate downstream signaling components in melanopsin or ipRGCs without affecting the rod/cone signaling system. Compounds that activate or inhibit light flux through ipRGCs should mimic pharmacological light or darkness and have potential applications. For instance, agonists would mimic light and provide a novel pharmaceutical intervention to elevate mood, while antagonists would mimic darkness and prevent light from inhibiting melatonin, thereby improving sleep.

### 4.3. Light management

The populace in industrialized nations is increasingly subjected to extended periods of artificial lighting, often well into the night. Moreover, the majority of hospitals and caregiving facilities maintain a state of perpetual illumination. While daylight-simulating light during daytime hours may offer benefits, nocturnal light exposure can disrupt circadian rhythms and associated physiological processes. This realization is prompting lighting manufacturers and architects to embrace dynamic lighting solutions for workplaces, caregiving environments, and residential settings.

Advances in our understanding of the light-eye-body axis now present opportunities to adjust lighting to promote optimal physical and mental health and performance. A newly developed, international standard provides a way of quantifying the influence of light on the intrinsically photosensitive, melanopsin-expressing, retinal neurons that mediate these effects which provides recommendations for lighting, based on an expert scientific consensus and expressed in an easily measured quantity (melanopic equivalent daylight illuminance (melaponic EDI)) defined within this standard 190. Throughout the daytime, the recommended minimum melanopic EDI is 250 lux at the eye measured in the vertical plane at approximately 1.2 m height. During the evening, starting at least 3 hours before bedtime, the recommended maximum melanopic EDI is 10 lux. The sleep environment should be as dark as possible. The recommended maximum ambient melanopic EDI is 1 lux.

Despite the potential to harness melanopsin function for human health improvements, significant challenges persist. Although melanopsin is retina-expressed and thus a subject of vision science, the primary consequences of disrupted melanopsin signaling are likely to manifest in sleep disturbances, mood disorders, and metabolic effects, which extend beyond the realm of vision science. This complexity calls for a nuanced approach that transcends traditional boundaries, engaging with disciplines such as chronobiology and endocrinology, to fully appreciate the implications of melanopsin signaling on human health and disease.

### 4.4. Light therapy

Clinical evidence supports the beneficial effects of light therapy for a variety of conditions, including depression, cognitive dysfunction, chronic pain, and sleep disorders 191. However, the unclear mechanisms of action have posed challenges for the clinical application of light therapy. Research of the light-eye-body axis can provide a deeper understanding of the mechanisms behind the therapeutic effects of light therapy.

Summarizing the aforementioned studies, we find that the effects of light therapy are associated with the wavelength, intensity, and duration of light exposure (Table. 1). For instance, strong light exposure during the day has antidepressant effects, while irregular light exposure at night may increase depression. Establishing new paradigms for phototherapy is crucial, as it can effectively activate specific visual pathways based on their unique photoresponses. This is essential for developing novel treatment strategies targeting the visual system to improve light therapy.

## 5. Conclusion and prospects

In this review, we pioneer a comprehensive discussion on the light-eye-body axis, a system where light, through the eyes and downstream neural pathways, regulates the entire body. The process of light signal transduction is primarily mediated by ipRGCs. We revisit the history of research from the discovery of ipRGCs, exploring their classification and functions, to their projections to various brain regions. Moreover, we analyze how light can influence a multitude of normal physiological functions and diseases in humans, as well as the mechanisms involved. Compared to other organs, the eye, with its immune privilege, constitutes a relatively independent system. However, its unique structure makes it the primary site for light perception and signal transduction. Traditional views held that there were two types of photoreceptors: rods and cones, responsible for image vision formation. The discovery of ipRGCs disrupted this balance and sparked a series of studies. ipRGCs not only receive input signals from rods and cones but can also detect light through melanopsin, a special protein they express, and project light signals to different brain areas, triggering various physiological effects. This structural basis is why ipRGCs are primarily responsible for non-image-forming vision. Existing research has demonstrated that under the influence of light signals, the eyes can control circadian rhythms and sleep, glucose metabolism, body temperature, mood, and cognition through various neural pathways. Light can also impact the human body under pathological conditions, including the development of myopia and photophobia. Utilizing the beneficial aspects of light, phototherapy has made significant progress in treating depression and pain. And we have observed that the light-eye-body axis plays a pivotal role in phototherapy for pain relief and the mitigation of depressive symptoms.

**Figure 4:**
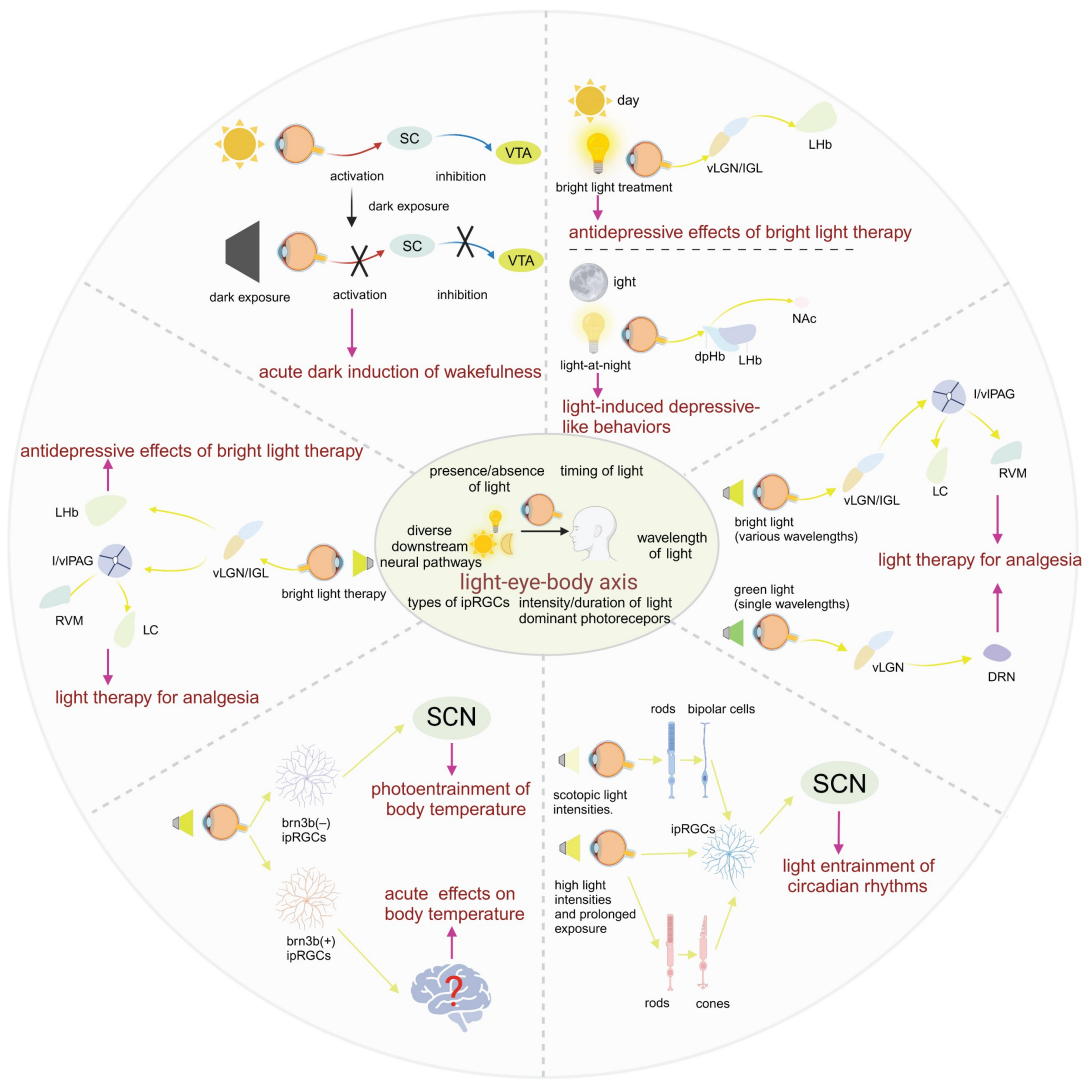
** Factors influencing the regulation of the light-eye**-**body axis.** SC: superior colliculus; VTA: ventral tegmental area; vLGN: ventral geniculate nucleus; IGL: intergeniculate leaflet; LHb: lateral habenula; dpHb: dorsal perihabenular nucleus; NAc: nucleus accumbens; l/vlPAG: lateral/ventral lateral parts of the periaqueductal gray area; RVM: rostral ventromedial medulla; LC: locus coeruleus; DRN: dorsal raphe nucleus; SCN: suprachiasmatic nucleus; IpRGCs: intrinsically photosensitive Retinal Ganglion Cells; RVM: rostral ventromedial medulla.

As discussed above, light exerts diverse and even contrasting effects on bodily functions through the eye, which are potentially influenced by the presence or absence of light, the timing of light exposure, the wavelength of light, the intensity of illumination, the duration of light exposure, the predominant photosensitive cells, the types of ipRGCs involved in pathway, and the diverse downstream neural pathways of ipRGCs (Table 1, Figure 4). For instance, light pulses induce sleep while dark pulses induce wakefulness. This exemplifies how existence or absence of light can significantly influence our sleep-wake behaver. Furthermore, morning bright light therapy can treat depression, whereas abnormal nighttime light exposure may lead to depression, due to the difference in light timing. The varying wavelengths of light also yield distinct effects: in light therapy for analgesia, green light, with its low tissue penetration, relieves pain only through the eye-nerve pathway. In contrast, red light, owing to its longer wavelength, can penetrate tissue directly for analgesia. Bright white light, encompassing all wavelengths of the visible spectrum, is also used for pain treatment, suggesting that its analgesic effect may be the result of numerous distinct wavelengths. Given the differing photosensitivity of various photoreceptive cells, they exhibit varying priorities under different lighting conditions. In the regulation of light entrainment of circadian rhythms, rod cells mediate circadian synchronization under low light intensities through the rod-bipolar pathway. However, under high light intensities and prolonged exposure, it is mediated by the rod-cone pathway and the inherent photosensitivity of ipRGCs, highlighting the impact of light intensity, exposure duration, and the predominant photosensitive cells in the light-eye-body axis. In the regulation of body temperature via the light-eye-body axis, Brn3b(-) ipRGCs project to the SCN, mediating circadian temperature synchronization, while Brn3b(+) ipRGCs project to other unknown brain regions, which are essential for acute temperature regulation. Their findings indicates that different types of ipRGCs involved in regulation also influence the effects of the light-eye-body axis. In conjunction with the positive effects of bright light therapy on depression and pain, it is evident that they are achieved through the activation of the retina-vLGN/IGL pathway by intense light. The activation of different downstream brain regions manifests different antidepressant and analgesic effects, demonstrating that diverse downstream neural pathways also influence the regulatory role of the light-eye-body axis on bodily functions. Overall, the light-eye-body axis exerts influence on various aspects of the body through distinct pathways, necessitating further exploration for its profound significance.

Looking ahead, we should value the concept of the light-eye-body axis and integrate it into scientific research, clinical practice, and daily life. First, for unexplored phototherapeutic mechanisms and physiological or pathological changes closely linked to light's influence, eye and particularly the ipRGCs within it may play a potential role. Second, for studies demonstrated that light regulated body through the eyes where specific pathways are yet to be discovered. The ipRGC pathways summarized in this review, combined with the functions of corresponding brain regions, could help identify possible directions. Third, deeper investigation into the light-eye-body axis and its controlling neural pathways is essential. Understanding the differences and connections will aid in managing the body's varied responses to different light stimuli. In previous discussions, we found that for intense light therapy, the ipRGC-vLGN/IGL pathway is shared for anti-depression and analgesia, differing only in downstream brain region projections, suggesting that intense light can activate protective mechanisms in humans, thus avoiding survival threats. Fourth, in clinical treatment, light therapy has been proven safe and effective for various diseases and is a cost-effective treatment method. A deeper understanding of the light-eye-body axis, as one of the key mechanisms of light therapy, will better guide the standardization and development of light therapy. Fifth, comprehending the light-eye-body axis concept can explain various phenomena in life and better protect our bodies. For example, the warm or cool tones of light may be due to different wavelengths of light stimulating brown adipose tissue's heat production through ipRGCs; nighttime light exposure significantly increases the risk of depression, and maintaining darkness in the bedroom at night can reduce the risk of depression 192. Sixthly, it is imperative to broaden our perspective beyond the acknowledged significance of neural pathways in the light-eye-body axis. The role of direct secretions from the eyes to the organism warrants equal attention. Recent studies suggest that retinal oxidative stress may influence the secretion of soluble macromolecules from the retina, potentially triggering chronic circadian rhythm disruptions and a systemic reduction in antioxidant defenses. This, in turn, could lead to oxidative stress in the liver 193. Seventh, as an integral component of the central nervous system, the retina shares numerous similarities with the brain in terms of neural signal transmission and processing. The retina offers unique advantages as research subject due to its relatively simple cellular structure and well-defined layers, providing a window into the complex behaviors and diseases of the brain. The disruption of ipRGCs is observed in a multitude of neurodegenerative diseases, such as Alzheimer's disease 194, Parkinson's disease 195, Huntington's disease 196, glaucoma 197, and diabetes 198 which suggests that ipRGCs, as key molecules in the retinal photoreceptive axis, may serve as a valuable biomarker for the early detection of these conditions.

Despite the vigorous ongoing research and notable achievements in the light-eye-body axis field, we must still focus on its shortcomings. First, many studies report flaws in ipRGC ablation techniques. When using SAP to ablate ipRGCs, it preferentially targets M1 to M3 types, leaving M4 to M6 types largely intact 22. Future work should employ more effective techniques to thoroughly ablate all ipRGC subtypes. Secondly, we should also pay attention to the non-image-forming visual effects of rod/cone photoreceptor light signal inputs. A review of the literature reveals that while most light-eye-body axis-related studies focus on ipRGCs. But it is cones rather than ipRGCs that play a key role in the mechanism of green light analgesia 171. Thirdly, although the mouse model is widely used due to its conservation with humans in ipRGC and many visual pathways, its limitations must be acknowledged. Humans are diurnal while mice are nocturnal; light exposure during the day or night increases human alertness, but for mice, nighttime light induces sleep, likely due to differences in the light signal neural pathways between diurnal and nocturnal animals 56. Additionally, mice are less suitable for higher cognitive function experiments. Utilizing the advantages and understanding the limitations of the mouse model will enable targeted exploration in future experiments. Fourthly, the majority of current research findings do not universally apply to group phenomena. For instance, there is a noticeable lack of studies on the impact of gender differences in animal experiments. In human research, the focus is often on younger, healthier participants with higher levels of education. However, there is less research on older populations and individuals with cognitive impairments. Studying these groups is crucial for the development and improvement of light therapy 131. Fifthly, few researchers have ventured into the exploration of the direct inter-organ communication through the light-eye-body axis via immune system and cytokines within the humoral circulation 199. This approach is a more straightforward and efficient method compared to the intricate neural pathways that are often the subject of study 200. Lastly, we should value the concept of the light-eye-body axis and conduct in-depth research to more comprehensively assess its role and value.

## Figures and Tables

**Table 1:** Summary of studies on the effects and mechanisms of light on migraine, depression and pain

Color	Subjects	Illumination time	Intensity	Symptom	Effect	Mechanism of Action	Study
White (390~760nm)	Human	2 seconds	50-20,000 lx	Migraine	↑	Visual system-Trigeminal and cervical pain perception thresholds decreased	Influence of intense light stimulation on trigeminal and cervical pain perception thresholds
	Rat	30min	2,000 lx	Migraine	↑	Retina-trigeminal caudal/cervical cord+nucleus tractus solitarius	Bright light produces Fos-positive neurons in caudal trigeminal brainstem
	Rat	10 sec	50,000 lx	Migraine	↑	IpRGCs-dura-sensitive thalamocortical neurons	A neural mechanism for exacerbation of headache by light
	Mouse	2h	3,000 lx	Excitatory effect of pain-related stimuli	↓	Retinal-vLGN/IGL-l/vlPAG	A visual circuit related to the periaqueductal gray area for the antinociceptive effects of bright light treatment
	Human	3 min	1-100 cd/m2	Migraine	↑	Cone-thalamic neurons-cortex	Migraine photophobia originating in cone-driven retinal pathways
Blue (450~500nm)	Human	3 min	1-100 cd/m2	Migraine	↑	Cone-thalamic neurons-cortex	Migraine photophobia originating in cone-driven retinal pathways
	Rat	12h	20lx	Depression	↓	IpRGC-DRN/amygdala	Antidepressant Effect of Blue Light on Depressive Phenotype in Light-Deprived Male Rats
	Rat	10 min	1300 lx	Depression	↓	IpRGCs-unknown pathway	Blue but not red light stimulation in the dark has antidepressant effect in behavioral despair
	Mouse	2 h	400 lx	Depression	↑	IpRGCs-dpHb-NAc	A circadian rhythm-gated subcortical pathway for nighttime-light-induced depressive-like behaviors in mice
	Mouse	2h	3,000 Lux	Depression	↓	M4 ipRGCs-vLGN/IGL-LHb	A Visual Circuit Related to Habenula Underlies the Antidepressive Effects of Light Therapy
Green (500~565nm)	Human	3 min	1-100 cd/m2	Migraine	↓	Cone-unkonwn pathway	Migraine photophobia originating in cone-driven retinal pathways
	Mouse	8 h	10 lx	Thermal hyperalgesia and mechanical allodynia	↓	Cone-vLGN-DRN	Green light analgesia in mice is mediated by visual activation of enkephalinergic neurons in the ventrolateral geniculate nucleus
	Rat	8 h	4 lux	Chronic pain	↓	Visual system-Descending pain inhibitory pathways from the RVM;Increased spinal cord expression of enkephalins	Long-lasting antinociceptive effects of green light in acute and chronic pain in rats
Amber 590nm	Human	3 min	1-100 cd/m2	Migraine	↑	Cone-thalamic neurons-cortex	Migraine photophobia originating in cone-driven retinal pathways
Red (625~740nm)	Human	3 min	1-100 cd/m2	Migraine	↑	Cone-unkonwn pathway	Migraine photophobia originating in cone-driven retinal pathways
	Rat	8 hr	50 lx	Thermal hyperalgesia and mechanical allodynia	↑	Visual system-RVM	Development and Characterization of An Injury-free Model of Functional Pain in Rats by Exposure to Red Light

## Abbreviations

RGCs: retinal ganglion cells
ipRGCs: intrinsically photosensitive retinal ganglion cells
IPL: inner plexiform layer
SCN: suprachiasmatic nucleus
LHb: lateral habenula
pSON: perisupraoptic nucleus
VMH: ventromedial nucleus
SC: superior colliculus
dLGN: dorsal geniculate nucleus
OPN: olivary pretectal nucleus
VTA: ventral tegmental area
POA: preoptic area
NREM: non-rapid eye movement
TMN: tuberomammillary
LH: lateral hypothalamus
DRN: dorsal raphe nucleus
REM: rapid eye movement
SON: supraoptic nucleus
BAT: brown adipose tissue
dLAN: dim light at night
PHb: perihabenular nucleus
vmPFC: ventromedial prefrontal cortex
MDD: major depressive disorders
NAc: nucleus accumbens
CeA: central amygdala
SAP: saporin
vLGN: ventral geniculate nucleus
PVN: paraventricular nucleus
HF: hair follicles
HFSC: hair follicle stem cells
α-MSH: alpha-melanocyte-stimulating hormone
CIB: contagious itch behavior
GRPR: gastrin-releasing peptide receptor
PVT: paraventricular nucleus of the thalamus
IGL: intergeniculate leaflet
Re: reunion nucleus
BLT: bright light therapy
LAN: light at night
MDD: major depressive disorder
dpHb: dorsal perihabenular nucleus
RLEDs: red light-emitting diodes
RVM: ventromedial medulla
l/vlPAG: lateral and ventral lateral parts of the periaqueductal gray area
LC: locus coeruleus
AL: axial length

## References

[1] Hu J, Shi Y, Zhang J, Huang X, Wang Q, Zhao H (2022). Melanopsin retinal ganglion cells mediate light-promoted brain development. Cell.

[2] Meng J-J, Shen J-W, Li G, Ouyang C-J, Hu J-X, Li Z-S (2023). Light modulates glucose metabolism by a retina-hypothalamus-brown adipose tissue axis. Cell.

[3] Cheung N, Mitchell P, Wong TY (2010). Diabetic retinopathy. Lancet.

[4] Bahn RS (2010). Graves' ophthalmopathy. N Engl J Med.

[5] Wu C-L S, Cioanca A V, Gelmi M C (2023). The multifunctional human ocular melanocortin system. Prog Retin Eye Res.

[6] Lei T, Hua H, Du H, Xia J, Xu D, Liu W (2024). Molecular mechanisms of artificial light at night affecting circadian rhythm disturbance. Arch Toxicol.

[7] Xiao N, Xu S, Li Z-K, Tang M, Mao R, Yang T (2023). A single photoreceptor splits perception and entrainment by cotransmission. Nature.

[8] Ingram NT, Sampath AP, Fain GL (2016). Why are rods more sensitive than cones?. J Physiol.

[9] Fu Y, Zhong H, Wang M-HH, Luo D-G, Liao H-W, Maeda H (2005). Intrinsically photosensitive retinal ganglion cells detect light with a vitamin A-based photopigment, melanopsin. Proc Natl Acad Sci U S A.

[10] Nagata E, Takao M, Toriumi H, Suzuki M, Fujii N, Kohara S (2024). Hypersensitivity of Intrinsically Photosensitive Retinal Ganglion Cells in Migraine Induces Cortical Spreading Depression. Int J Mol Sci.

[11] Huang W, Xu Q, Su J, Tang L, Hao Z-Z, Xu C (2022). Linking transcriptomes with morphological and functional phenotypes in mammalian retinal ganglion cells. Cell Reports.

[12] Wang AYM, Kulkarni MM, McLaughlin AJ, Gayet J, Smith BE, Hauptschein M (2023). An ON-type direction-selective ganglion cell in primate retina. Nature.

[13] Sanes JR, Masland RH (2015). The Types of Retinal Ganglion Cells: Current Status and Implications for Neuronal Classification. Annu Rev Neurosci.

[14] Liu J, Sanes JR (2017). Cellular and Molecular Analysis of Dendritic Morphogenesis in a Retinal Cell Type That Senses Color Contrast and Ventral Motion. J Neurosci.

[15] Berson DM, Dunn FA, Takao M (2002). Phototransduction by retinal ganglion cells that set the circadian clock. Science.

[16] Do MTH, Kang SH, Xue T, Zhong H, Liao H-W, Bergles DE (2009). Photon capture and signalling by melanopsin retinal ganglion cells. Nature.

[17] Euler T, Haverkamp S, Schubert T, Baden T (2014). Retinal bipolar cells: elementary building blocks of vision. Nat Rev Neurosci.

[18] Berson DM, Castrucci AM, Provencio I (2010). Morphology and mosaics of melanopsin-expressing retinal ganglion cell types in mice. J Comp Neurol.

[19] Baver SB, Pickard GE, Sollars PJ, Pickard GE (2008). Two types of melanopsin retinal ganglion cell differentially innervate the hypothalamic suprachiasmatic nucleus and the olivary pretectal nucleus. European Journal of Neuroscience.

[20] Li JY, Schmidt TM (2018). Divergent projection patterns of M1 ipRGC subtypes. J Comp Neurol.

[21] Schmidt TM, Kofuji P (2009). Functional and morphological differences among intrinsically photosensitive retinal ganglion cells. J Neurosci.

[22] Müller LP de S, Do MTH, Yau K-W, He S, Baldridge WH (2010). Tracer coupling of intrinsically photosensitive retinal ganglion cells to amacrine cells in the mouse retina. J Comp Neurol.

[23] Schmidt TM, Kofuji P (2011). Structure and function of bistratified intrinsically photosensitive retinal ganglion cells in the mouse. J Comp Neurol.

[24] Ecker JL, Dumitrescu ON, Wong KY, Alam NM, Chen S-K, LeGates T (2010). Melanopsin-expressing retinal ganglion-cell photoreceptors: cellular diversity and role in pattern vision. Neuron.

[25] Viney TJ, Balint K, Hillier D, Siegert S, Boldogkoi Z, Enquist LW (2007). Local retinal circuits of melanopsin-containing ganglion cells identified by transsynaptic viral tracing. Curr Biol.

[26] Stabio ME, Sabbah S, Quattrochi LE, Ilardi MC, Fogerson PM, Leyrer ML (2018). The M5 Cell: A Color-Opponent Intrinsically Photosensitive Retinal Ganglion Cell. Neuron.

[27] Quattrochi LE, Stabio ME, Kim I, Ilardi MC, Michelle Fogerson P, Leyrer ML (2019). The M6 cell: A small-field bistratified photosensitive retinal ganglion cell. J Comp Neurol.

[28] Stephenson KM, Schroder CM, Bertschy G, Bourgin P (2012). Complex interaction of circadian and non-circadian effects of light on mood: shedding new light on an old story. Sleep Med Rev.

[29] LeGates TA, Fernandez DC, Hattar S (2014). Light as a central modulator of circadian rhythms, sleep and affect. Nat Rev Neurosci.

[30] Hattar S, Kumar M, Park A, Tong P, Tung J, Yau K-W (2006). Central projections of melanopsin-expressing retinal ganglion cells in the mouse. Journal of Comparative Neurology.

[31] Estevez ME, Fogerson PM, Ilardi MC, Borghuis BG, Chan E, Weng S (2012). Form and function of the M4 cell, an intrinsically photosensitive retinal ganglion cell type contributing to geniculocortical vision. J Neurosci.

[32] Weng S, Estevez ME, Berson DM (2013). Mouse ganglion-cell photoreceptors are driven by the most sensitive rod pathway and by both types of cones. PLoS One.

[33] Hattar S, Liao H-W, Takao M, Berson DM, Yau K-W (2002). Melanopsin-Containing Retinal Ganglion Cells: Architecture, Projections, and Intrinsic Photosensitivity. Science.

[34] Patterson SS, Neitz M, Neitz J (2022). S-cone circuits in the primate retina for non-image-forming vision. Semin Cell Dev Biol.

[35] Hattar S, Lucas RJ, Mrosovsky N, Thompson S, Douglas RH, Hankins MW (2003). Melanopsin and rod-cone photoreceptive systems account for all major accessory visual functions in mice. Nature.

[36] Liu A-L, Liu Y-F, Wang G, Shao Y-Q, Yu C-X, Yang Z (2022). The role of ipRGCs in ocular growth and myopia development. Sci Adv.

[37] Dumitrescu ON, Pucci FG, Wong KY, Berson DM (2009). Ectopic Retinal ON Bipolar Cell Synapses in the OFF Inner Plexiform Layer: Contacts with Dopaminergic Amacrine Cells and Melanopsin Ganglion Cells. The Journal of Comparative Neurology.

[38] Schmidt TM, Kofuji P (2011). Structure and Function of Bistratified Intrinsically Photosensitive Retinal Ganglion Cells in the Mouse. The Journal of comparative neurology.

[39] Schwartz GW, Okawa H, Dunn FA, Morgan JL, Kerschensteiner D, Wong RO (2012). The spatial structure of a nonlinear receptive field. Nature neuroscience.

[40] Güler AD, Ecker JL, Lall GS, Haq S, Altimus CM, Liao H-W (2008). Melanopsin cells are the principal conduits for rod-cone input to non-image-forming vision. Nature.

[41] Huang X, Huang P, Huang L, Hu Z, Liu X, Shen J (2021). A Visual Circuit Related to the Nucleus Reuniens for the Spatial-Memory-Promoting Effects of Light Treatment. Neuron.

[42] Fernandez DC, Fogerson PM, Ospri LL, Thomsen MB, Layne RM, Severin D (2018). Light affects mood and learning through distinct retina-brain pathways. Cell.

[43] Altimus CM, Güler AD, Villa KL, McNeill DS, LeGates TA, Hattar S (2008). Rods-cones and melanopsin detect light and dark to modulate sleep independent of image formation. Proc Natl Acad Sci U S A.

[44] Rupp AC, Ren M, Altimus CM, Fernandez DC, Richardson M, Turek F (2019). Distinct ipRGC subpopulations mediate light's acute and circadian effects on body temperature and sleep. Liberles S, Dulac C, Liberles S, Eds. eLife.

[45] Ibuka N, Inouye SI, Kawamura H (1977). Analysis of sleep-wakefulness rhythms in male rats after suprachiasmatic nucleus lesions and ocular enucleation. Brain Res.

[46] Fernandez DC, Chang Y-T, Hattar S, Chen S-K (2016). Architecture of retinal projections to the central circadian pacemaker. Proc Natl Acad Sci U S A.

[47] Panda S, Sato TK, Castrucci AM, Rollag MD, DeGrip WJ, Hogenesch JB (2002). Melanopsin (Opn4) requirement for normal light-induced circadian phase shifting. Science.

[48] Lucas RJ, Hattar S, Takao M, Berson DM, Foster RG, Yau K-W (2003). Diminished pupillary light reflex at high irradiances in melanopsin-knockout mice. Science.

[49] Altimus CM, Güler AD, Alam NM, Arman AC, Prusky GT, Sampath AP (2010). Rod photoreceptors drive circadian photoentrainment across a wide range of light intensities. Nat Neurosci.

[50] Göz D, Studholme K, Lappi DA, Rollag MD, Provencio I, Morin LP (2008). Targeted Destruction of Photosensitive Retinal Ganglion Cells with a Saporin Conjugate Alters the Effects of Light on Mouse Circadian Rhythms. PLoS One.

[51] Lupi D, Oster H, Thompson S, Foster RG (2008). The acute light-induction of sleep is mediated by OPN4-based photoreception. Nat Neurosci.

[52] Zhang Z, Liu W-Y, Diao Y-P, Xu W, Zhong Y-H, Zhang J-Y (2019). Superior Colliculus GABAergic Neurons Are Essential for Acute Dark Induction of Wakefulness in Mice. Current Biology.

[53] Kroeger D, Absi G, Gagliardi C, Bandaru SS, Madara JC, Ferrari LL (2018). Galanin neurons in the ventrolateral preoptic area promote sleep and heat loss in mice. Nat Commun.

[54] Cai P, Huang S-N, Lin Z-H, Wang Z, Liu R-F, Xiao W-H (2022). Regulation of wakefulness by astrocytes in the lateral hypothalamus. Neuropharmacology.

[55] Yu X, Li W, Ma Y, Tossell K, Harris JJ, Harding EC (2019). GABA and glutamate neurons in the VTA regulate sleep and wakefulness. Nat Neurosci.

[56] Zhang Z, Beier C, Weil T, Hattar S (2021). The retinal ipRGC-preoptic circuit mediates the acute effect of light on sleep. Nat Commun.

[57] Zhang Z, Wang H-J, Wang D-R, Qu W-M, Huang Z-L (2017). Red light at intensities above 10 lx alters sleep-wake behavior in mice. Light Sci Appl.

[58] Rillig MC, Antonovics J (2019). Microbial biospherics: The experimental study of ecosystem function and evolution. Proc Natl Acad Sci U S A.

[59] Geoghegan G, Simcox J (2023). SON-light activation of glucose regulation. Cell.

[60] Zheng R, Xin Z, Li M, Wang T, Xu M, Lu J (2023). Outdoor light at night in relation to glucose homoeostasis and diabetes in Chinese adults: a national and cross-sectional study of 98,658 participants from 162 study sites. Diabetologia.

[61] Stenvers DJ, Scheer FAJL, Schrauwen P, la Fleur SE, Kalsbeek A (2019). Circadian clocks and insulin resistance. Nat Rev Endocrinol.

[62] Fan X, Chen D, Wang Y, Tan Y, Zhao H, Zeng J (2022). Light intensity alters the effects of light-induced circadian disruption on glucose and lipid metabolism in mice. Am J Physiol Endocrinol Metab.

[63] Mogensen MF, English HB (1926). The Apparent Warmth of Colors. The American Journal of Psychology.

[64] Park Y-MM, White AJ, Jackson CL, Weinberg CR, Sandler DP (2019). Association of Exposure to Artificial Light at Night While Sleeping With Risk of Obesity in Women. JAMA Internal Medicine.

[65] Morris CJ, Yang JN, Garcia JI, Myers S, Bozzi I, Wang W (2015). Endogenous circadian system and circadian misalignment impact glucose tolerance via separate mechanisms in humans. Proc Natl Acad Sci U S A.

[66] Bedrosian TA, Fonken LK, Walton JC, Haim A, Nelson RJ (2011). Dim light at night provokes depression-like behaviors and reduces CA1 dendritic spine density in female hamsters. Psychoneuroendocrinology.

[67] LeGates TA, Altimus CM, Wang H, Lee H-K, Yang S, Zhao H (2012). Aberrant light directly impairs mood and learning through melanopsin-expressing neurons. Nature.

[68] Morin LP (2013). Neuroanatomy of the Extended Circadian Rhythm System. Exp Neurol.

[69] Meng C, Brandl F, Tahmasian M, Shao J, Manoliu A, Scherr M (2014). Aberrant topology of striatum's connectivity is associated with the number of episodes in depression. Brain.

[70] Shen X, Reus LM, Cox SR, Adams MJ, Liewald DC, Bastin ME (2017). Subcortical volume and white matter integrity abnormalities in major depressive disorder: findings from UK Biobank imaging data. Sci Rep.

[71] Kerestes R, Harrison BJ, Dandash O, Stephanou K, Whittle S, Pujol J (2014). Specific functional connectivity alterations of the dorsal striatum in young people with depression. Neuroimage Clin.

[72] Francis TC, Lobo MK (2017). Emerging role for nucleus accumbens medium spiny neuron subtypes in depression. Biol Psychiatry.

[73] Lazzerini Ospri L, Zhan JJ, Thomsen MB, Wang H, Komal R, Tang Q (2024). Light affects the prefrontal cortex via intrinsically photosensitive retinal ganglion cells. Sci Adv.

[74] Ziółkowska N, Chmielewska-Krzesińska M, Vyniarska A, Sienkiewicz W (2022). Exposure to Blue Light Reduces Melanopsin Expression in Intrinsically Photoreceptive Retinal Ganglion Cells and Damages the Inner Retina in Rats. Invest Ophthalmol Vis Sci.

[75] Ziółkowska N, Lewczuk B, Szyryńska N, Rawicka A, Vyniarska A (2023). Low-Intensity Blue Light Exposure Reduces Melanopsin Expression in Intrinsically Photosensitive Retinal Ganglion Cells and Damages Mitochondria in Retinal Ganglion Cells in Wistar Rats. Cells.

[76] Roh S-C, Park E-J, Shim M, Lee S-H (2016). EEG beta and low gamma power correlates with inattention in patients with major depressive disorder. J Affect Disord.

[77] Hammar Å, Årdal G (2009). Cognitive Functioning in Major Depression - A Summary. Front Hum Neurosci.

[78] Adhikari A (2014). Distributed circuits underlying anxiety. Front Behav Neurosci.

[79] Wang G, Liu Y-F, Yang Z, Yu C-X, Tong Q, Tang Y-L (2023). Short-term acute bright light exposure induces a prolonged anxiogenic effect in mice via a retinal ipRGC-CeA circuit. Sci Adv.

[80] Schmidt TM, Kofuji P (2010). Differential Cone Pathway Influence on Intrinsically Photosensitive Retinal Ganglion Cell Subtypes. The Journal of Neuroscience.

[81] Davis M (1992). The role of the amygdala in fear and anxiety. Annu Rev Neurosci.

[82] Tu DC, Zhang D, Demas J, Slutsky EB, Provencio I, Holy TE (2005). Physiologic diversity and development of intrinsically photosensitive retinal ganglion cells. Neuron.

[83] Wiesel TN, Hubel DH (1963). Single-cell responses in striate cortex of kittens deprived of vision in one eye. Journal of Neurophysiology.

[84] Espinosa JS, Stryker MP (2012). Development and Plasticity of the Primary Visual Cortex. Neuron.

[85] Andrabi M, Upton BA, Lang RA, Vemaraju S (2023). An Expanding Role for Nonvisual Opsins in Extraocular Light Sensing Physiology. Annual Review of Vision Science.

[86] Rao S, Chun C, Fan J, Kofron JM, Yang MB, Hegde RS (2013). A direct and melanopsin-dependent fetal light response regulates mouse eye development. Nature.

[87] Tufford AR, Onyak JR, Sondereker KB, Lucas JA, Earley AM, Mattar P (2018). Melanopsin Retinal Ganglion Cells Regulate Cone Photoreceptor Lamination in the Mouse Retina. Cell Reports.

[88] Kinane C, Calligaro H, Jandot A, Coutanson C, Haddjeri N, Bennis M (2023). Dopamine modulates the retinal clock through melanopsin-dependent regulation of cholinergic waves during development. BMC Biology.

[89] Chew KS, Renna JM, McNeill DS, Fernandez DC, Keenan WT, Thomsen MB (2017). A subset of ipRGCs regulates both maturation of the circadian clock and segregation of retinogeniculate projections in mice. Takahashi JS, Ed. eLife.

[90] D'Souza SP, Upton BA, Eldred KC, Glass I, Nayak G, Grover K (2024). Developmental control of rod number via a light-dependent retrograde pathway from intrinsically photosensitive retinal ganglion cells. Dev Cell.

[91] Ji BW, Sheth RU, Dixit PD, Tchourine K, Vitkup D (2020). Macroecological dynamics of gut microbiota. Nat Microbiol.

[92] Leone V, Gibbons SM, Martinez K, Hutchison AL, Huang EY, Cham CM (2015). Effects of diurnal variation of gut microbes and high fat feeding on host circadian clock function and metabolism. Cell Host Microbe.

[93] Liang F, Chen C-Y, Li Y-P, Ke Y-C, Ho E-P, Jeng C-F (2022). Early Dysbiosis and Dampened Gut Microbe Oscillation Precede Motor Dysfunction and Neuropathology in Animal Models of Parkinson's Disease. J Parkinsons Dis.

[94] Reitmeier S, Kiessling S, Clavel T, List M, Almeida EL, Ghosh TS (2020). Arrhythmic Gut Microbiome Signatures Predict Risk of Type 2 Diabetes. Cell Host Microbe.

[95] Lee C, Liang F, Lee I, Lu T, Shan Y, Jeng C (2022). External light-dark cycle shapes gut microbiota through intrinsically photosensitive retinal ganglion cells. EMBO Rep.

[96] McGarvey EL, Baum LD, Pinkerton RC, Rogers LM (2001). Psychological sequelae and alopecia among women with cancer. Cancer Pract.

[97] Chen C-L, Huang W-Y, Wang EHC, Tai K-Y, Lin S-J (2020). Functional complexity of hair follicle stem cell niche and therapeutic targeting of niche dysfunction for hair regeneration. Journal of Biomedical Science.

[98] Hsu Y-C, Pasolli HA, Fuchs E (2011). Dynamics Between Stem Cells, Niche and Progeny in the Hair Follicle. Cell.

[99] Hu X-M, Li Z-X, Zhang D-Y, Yang Y-C, Fu S, Zhang Z-Q (2021). A systematic summary of survival and death signalling during the life of hair follicle stem cells. Stem Cell Res Ther.

[100] Ali N, Zirak B, Rodriguez RS, Pauli ML, Truong H-A, Lai K (2017). Regulatory T cells in Skin Facilitate Epithelial Stem Cell Differentiation. Cell.

[101] Fan SM-Y, Chang Y-T, Chen C-L, Wang W-H, Pan M-K, Chen W-P (2018). External light activates hair follicle stem cells through eyes via an ipRGC-SCN-sympathetic neural pathway. Proc Natl Acad Sci U S A.

[102] Zhang B, Ma S, Rachmin I, He M, Baral P, Choi S (2020). Hyperactivation of sympathetic nerves drives depletion of melanocyte stem cells. Nature.

[103] Niijima A, Nagai K, Nagai N, Akagawa H (1993). Effects of light stimulation on the activity of the autonomic nerves in anesthetized rats. Physiol Behav.

[104] Filadelfi AMC, Vieira A, Louzada FM (2005). Circadian rhythm of physiological color change in the amphibian Bufo ictericus under different photoperiods. Comp Biochem Physiol A Mol Integr Physiol.

[105] Auerswald L, Freier U, Lopata A, Meyer B (2008). Physiological and morphological colour change in Antarctic krill, Euphausia superba: a field study in the Lazarev Sea. J Exp Biol.

[106] Oshima N (2001). Direct reception of light by chromatophores of lower vertebrates. Pigment Cell Res.

[107] Bertolesi GE, Hehr CL, McFarlane S (2015). Melanopsin photoreception in the eye regulates light-induced skin colour changes through the production of α-MSH in the pituitary gland. Pigment Cell & Melanoma Research.

[108] Bertolesi GE, Hehr CL, Munn H, McFarlane S (2016). Two light-activated neuroendocrine circuits arising in the eye trigger physiological and morphological pigmentation. Pigment Cell Melanoma Res.

[109] Itti L, Koch C (2001). Computational modelling of visual attention. Nat Rev Neurosci.

[110] de Waal FBM, Preston SD (2017). Mammalian empathy: behavioural manifestations and neural basis. Nat Rev Neurosci.

[111] Prochazkova E, Kret ME (2017). Connecting minds and sharing emotions through mimicry: A neurocognitive model of emotional contagion. Neurosci Biobehav Rev.

[112] Yu Y-Q, Barry DM, Hao Y, Liu X-T, Chen Z-F (2017). Molecular and neural basis of contagious itch behavior in mice. Science.

[113] Gao F, Ma J, Yu Y-Q, Gao X-F, Bai Y, Sun Y (2022). A non-canonical retina-ipRGCs-SCN-PVT visual pathway for mediating contagious itch behavior. Cell Rep.

[114] Chartrand TL, Lakin JL (2013). The antecedents and consequences of human behavioral mimicry. Annu Rev Psychol.

[115] Langford DJ, Crager SE, Shehzad Z, Smith SB, Sotocinal SG, Levenstadt JS (2006). Social modulation of pain as evidence for empathy in mice. Science.

[116] Panksepp JB, Lahvis GP (2011). Rodent Empathy and Affective Neuroscience. Neurosci Biobehav Rev.

[117] van Jaarsveld B, Bennett NC, Hart DW, Oosthuizen MK (2019). Locomotor activity and body temperature rhythms in the Mahali mole-rat (C. h. mahali): The effect of light and ambient temperature variations. J Therm Biol.

[118] Badia P, Myers B, Boecker M, Culpepper J, Harsh JR (1991). Bright light effects on body temperature, alertness, EEG and behavior. Physiol Behav.

[119] Cajochen C, Münch M, Kobialka S, Kräuchi K, Steiner R, Oelhafen P (2005). High sensitivity of human melatonin, alertness, thermoregulation, and heart rate to short wavelength light. J Clin Endocrinol Metab.

[120] Panda S, Nayak SK, Campo B, Walker JR, Hogenesch JB, Jegla T (2005). Illumination of the melanopsin signaling pathway. Science.

[121] Wright KP, Hull JT, Czeisler CA (2002). Relationship between alertness, performance, and body temperature in humans. Am J Physiol Regul Integr Comp Physiol.

[122] Csábi E, Benedek P, Janacsek K, Zavecz Z, Katona G, Nemeth D (2015). Declarative and Non-declarative Memory Consolidation in Children with Sleep Disorder. Front Hum Neurosci.

[123] Warthen DM, Wiltgen BJ, Provencio I (2011). Light enhances learned fear. Proceedings of the National Academy of Sciences of the United States of America.

[124] Soler JE, Robison AJ, Núñez AA, Yan L (2018). Light modulates hippocampal function and spatial learning in a diurnal rodent species: A study using male nile grass rat (Arvicanthis niloticus). Hippocampus.

[125] Hasan S, Tam SKE, Foster RG, Vyazovskiy VV, Bannerman DM, Peirson SN (2021). Modulation of recognition memory performance by light and its relationship with cortical EEG theta and gamma activities. Biochem Pharmacol.

[126] Cum M, Santiago Pérez JA, Wangia E, Lopez N, Wright ES, Iwata RL (2024). A systematic review and meta-analysis of how social memory is studied. Sci Rep.

[127] Huang Y-F, Liao P-Y, Yu J-H, Chen S-K (2023). Light disrupts social memory via a retina-to-supraoptic nucleus circuit. EMBO Rep.

[128] Ferguson JN, Aldag JM, Insel TR, Young LJ (2001). Oxytocin in the medial amygdala is essential for social recognition in the mouse. J Neurosci.

[129] Devarajan K, Marchant EG, Rusak B (2005). Circadian and light regulation of oxytocin and parvalbumin protein levels in the ciliated ependymal layer of the third ventricle in the C57 mouse. Neuroscience.

[130] Maren S (2001). Neurobiology of Pavlovian fear conditioning. Annu Rev Neurosci.

[131] Mahoney HL (2024). The cognitive impact of light: illuminating ipRGC circuit mechanisms. Nature Reviews Neuroscience.

[132] Alkozei A, Smith R, Dailey NS, Bajaj S, Killgore WDS (2017). Acute exposure to blue wavelength light during memory consolidation improves verbal memory performance. PLoS One.

[133] Chantranupong L, Sabatini BL (2018). Sunlight Brightens Learning and Memory. Cell.

[134] Mure LS, Vinberg F, Hanneken A, Panda S (2019). Functional diversity of human intrinsically photosensitive retinal ganglion cells. Science.

[135] Wu Y, Hallett M (2017). Photophobia in neurologic disorders. Transl Neurodegener.

[136] Kowacs P, Piovesan E, Werneck L, Tatsui C, Lange M, Ribas L (2001). Influence of Intense Light Stimulation on Trigeminal and Cervical Pain Perception Thresholds. Cephalalgia.

[137] Noseda R, Kainz V, Jakubowski M, Gooley JJ, Saper CB, Digre K (2010). A neural mechanism for exacerbation of headache by light. Nat Neurosci.

[138] Okamoto K, Thompson R, Tashiro A, Chang Z, Bereiter DA (2009). Bright light produces Fos-positive neurons in caudal trigeminal brainstem. Neuroscience.

[139] Maleki N, Becerra L, Upadhyay J, Burstein R, Borsook D (2012). Direct optic nerve pulvinar connections defined by diffusion MR tractography in humans: implications for photophobia. Hum Brain Mapp.

[140] Main A, Vlachonikolis I, Dowson A (2000). The wavelength of light causing photophobia in migraine and tension-type headache between attacks. Headache.

[141] Zaidi FH, Hull JT, Peirson SN, Wulff K, Aeschbach D, Gooley JJ (2007). Short-Wavelength Light Sensitivity of Circadian, Pupillary, and Visual Awareness in Humans Lacking an Outer Retina. Curr Biol.

[142] Noseda R, Bernstein CA, Nir R-R, Lee AJ, Fulton AB, Bertisch SM (2016). Migraine photophobia originating in cone-driven retinal pathways. Brain.

[143] Strauss ED, Schloss KB, Palmer SE (2013). Color preferences change after experience with liked/disliked colored objects. Psychon Bull Rev.

[144] Garbazza C, Cirignotta F, D'Agostino A, Cicolin A, Hackethal S, Wirz-Justice A (2022). Sustained remission from perinatal depression after bright light therapy: A pilot randomised, placebo-controlled trial. Acta Psychiatr Scand.

[145] Sit DK, McGowan J, Wiltrout C, Diler RS, Dills JJ, Luther J (2018). Adjunctive Bright Light Therapy for Bipolar Depression: A Randomized Double-Blind Placebo-Controlled Trial. Am J Psychiatry.

[146] Bedrosian TA, Nelson RJ (2013). Influence of the modern light environment on mood. Mol Psychiatry.

[147] Zielinska-Dabkowska KM (2018). Make lighting healthier. Nature.

[148] Huang L, Xi Y, Peng Y, Yang Y, Huang X, Fu Y (2019). A Visual Circuit Related to Habenula Underlies the Antidepressive Effects of Light Therapy. Neuron.

[149] İyilikci O, Aydin E, Canbeyli R (2009). Blue but not red light stimulation in the dark has antidepressant effect in behavioral despair. Behavioural Brain Research.

[150] Meng Q, Jiang J, Hou X, Jia L, Duan X, Zhou W (2020). Antidepressant Effect of Blue Light on Depressive Phenotype in Light-Deprived Male Rats. Journal of Neuropathology & Experimental Neurology.

[151] Ostrin LA (2019). Ocular and systemic melatonin and the influence of light exposure. Clin Exp Optom.

[152] An K, Zhao H, Miao Y, Xu Q, Li Y-F, Ma Y-Q (2020). A circadian rhythm-gated subcortical pathway for nighttime-light-induced depressive-like behaviors in mice. Nat Neurosci.

[153] Y Y, Y C, K S, Y D, Z N, S M (2018). Ketamine blocks bursting in the lateral habenula to rapidly relieve depression. Nature.

[154] Falchi F, Cinzano P, Duriscoe D, Kyba CCM, Elvidge CD, Baugh K (2016). The new world atlas of artificial night sky brightness. Sci Adv.

[155] Lam RW, Levitt AJ, Levitan RD, Michalak EE, Cheung AH, Morehouse R (2016). Efficacy of Bright Light Treatment, Fluoxetine, and the Combination in Patients With Nonseasonal Major Depressive Disorder: A Randomized Clinical Trial. JAMA Psychiatry.

[156] Rutten S, Vriend C, Smit JH, Berendse HW, Someren EJW van, Hoogendoorn AW (2019). Bright light therapy for depression in Parkinson disease: A randomized controlled trial. Neurology.

[157] Figueiro MG, Plitnick B, Roohan C, Sahin L, Kalsher M, Rea MS (2019). Effects of a Tailored Lighting Intervention on Sleep Quality, Rest-Activity, Mood, and Behavior in Older Adults With Alzheimer Disease and Related Dementias: A Randomized Clinical Trial. J Clin Sleep Med.

[158] Kim W-H, Joa K-L, Kim C-B, Lee H-S, Kang S-G, Jung H-Y (2022). The Effect of Bright Light Therapy on Sleep and Quality of Life in Patients With Poststroke Insomnia. Psychosom Med.

[159] Martin L, Porreca F, Mata EI, Salloum M, Goel V, Gunnala P (2021). Green Light Exposure Improves Pain and Quality of Life in Fibromyalgia Patients: A Preliminary One-Way Crossover Clinical Trial. Pain Med.

[160] Martin LF, Patwardhan AM, Jain SV, Salloum MM, Freeman J, Khanna R (2021). Evaluation of green light exposure on headache frequency and quality of life in migraine patients: A preliminary one-way cross-over clinical trial. Cephalalgia.

[161] Leichtfried V, Matteucci Gothe R, Kantner-Rumplmair W, Mair-Raggautz M, Bartenbach C, Guggenbichler H (2014). Short-term effects of bright light therapy in adults with chronic nonspecific back pain: a randomized controlled trial. Pain Med.

[162] Pigatto GR, Quinteiro MHS, Nunes-de-Souza RL, Coimbra NC, Parizotto NA (2020). Low-Intensity Photobiomodulation Decreases Neuropathic Pain in Paw Ischemia-Reperfusion and Spared Nervus Ischiadicus Injury Experimental Models. Pain Pract.

[163] DE Oliveira MF, Johnson DS, Demchak T, Tomazoni SS, Leal-Junior EC (2022). Low-intensity LASER and LED (photobiomodulation therapy) for pain control of the most common musculoskeletal conditions. Eur J Phys Rehabil Med.

[164] Nir R-R, Lee AJ, Huntington S, Noseda R, Bernstein CA, Fulton AB (2018). Color-selective photophobia in ictal vs interictal migraineurs and in healthy controls. PAIN.

[165] Khanna R, Patwardhan A, Yang X, Li W, Cai S, Ji Y (2019). Development and Characterization of An Injury-free Model of Functional Pain in Rats by Exposure to Red Light. J Pain.

[166] Zhang Y, Zhao S, Rodriguez E, Takatoh J, Han B-X, Zhou X (2015). Identifying local and descending inputs for primary sensory neurons. J Clin Invest.

[167] Cheng K, Martin LF, Slepian MJ, Patwardhan AM, Ibrahim MM (2021). Mechanisms and Pathways of Pain Photobiomodulation: A Narrative Review. J Pain.

[168] Ibrahim MM, Patwardhan A, Gilbraith KB, Moutal A, Yang X, Chew LA (2017). Long-lasting antinociceptive effects of green light in acute and chronic pain in rats. Pain.

[169] Wu X-Q, Tan B, Du Y, Yang L, Hu T-T, Ding Y-L (2023). Glutamatergic and GABAergic neurons in the vLGN mediate the nociceptive effects of green and red light on neuropathic pain. Neurobiology of Disease.

[170] Hu Z, Mu Y, Huang L, Hu Y, Chen Z, Yang Y (2022). A visual circuit related to the periaqueductal gray area for the antinociceptive effects of bright light treatment. Neuron.

[171] Tang Y-L, Liu A-L, Lv S-S, Zhou Z-R, Cao H, Weng S-J (2022). Green light analgesia in mice is mediated by visual activation of enkephalinergic neurons in the ventrolateral geniculate nucleus. Sci Transl Med.

[172] Burns JW, Gerhart J, Rizvydeen M, Kimura M, Burgess HJ (2020). Morning Bright Light Treatment for Chronic Low Back Pain: Potential Impact on the Volatility of Pain, Mood, Function, and Sleep. Pain Med.

[173] Vandewalle G, Maquet P, Dijk D-J (2009). Light as a modulator of cognitive brain function. Trends in Cognitive Sciences.

[174] Yates D (2022). Shining a light on pain. Nat Rev Neurosci.

[175] Balbinot G, Schuch CP, Nascimento PS do, Lanferdini FJ, Casanova M, Baroni BM (2021). Photobiomodulation Therapy Partially Restores Cartilage Integrity and Reduces Chronic Pain Behavior in a Rat Model of Osteoarthritis: Involvement of Spinal Glial Modulation. Cartilage.

[176] Schmidt TM, Alam NM, Chen S, Kofuji P, Li W, Prusky GT (2014). A role for melanopsin in alpha retinal ganglion cells and contrast detection. Neuron.

[177] Hansen MM, Jones R, Tocchini K (2017). Shinrin-Yoku (Forest Bathing) and Nature Therapy: A State-of-the-Art Review. Int J Environ Res Public Health.

[178] Gil S, Le Bigot L (2014). Seeing life through positive-tinted glasses: color-meaning associations. PLoS One.

[179] Vidal-Sanz M, Galindo-Romero C, Valiente-Soriano FJ, Nadal-Nicolás FM, Ortin-Martinez A, Rovere G (2017). Shared and Differential Retinal Responses against Optic Nerve Injury and Ocular Hypertension. Front Neurosci.

[180] Reutrakul S, Park JC, McAnany JJ, Chau FY, Danielson KK, Prasad B (2024). Dysregulated 24 h melatonin secretion associated with intrinsically photosensitive retinal ganglion cell function in diabetic retinopathy: a cross-sectional study. Diabetologia.

[181] Rukmini AV, Milea D, Baskaran M, How AC, Perera SA, Aung T (2015). Pupillary Responses to High-Irradiance Blue Light Correlate with Glaucoma Severity. Ophthalmology.

[182] Ahn S-H, Suh J-S, Lim G-H, Kim T-J (2023). The Potential Effects of Light Irradiance in Glaucoma and Photobiomodulation Therapy. Bioengineering.

[183] Groff ML, Choi B, Lin T, Mcllraith I, Hutnik C, Malvankar-Mehta MS (2023). Anxiety, depression, and sleep-related outcomes of glaucoma patients: systematic review and meta-analysis. Can J Ophthalmol.

[184] Holden BA, Fricke TR, Wilson DA, Jong M, Naidoo KS, Sankaridurg P (2016). Global Prevalence of Myopia and High Myopia and Temporal Trends from 2000 through 2050. Ophthalmology.

[185] Wallman J, Winawer J (2004). Homeostasis of eye growth and the question of myopia. Neuron.

[186] Chakraborty R, Landis EG, Mazade R, Yang V, Strickland R, Hattar S (2022). Melanopsin modulates refractive development and myopia. Exp Eye Res.

[187] Prigge CL, Yeh P-T, Liou N-F, Lee C-C, You S-F, Liu L-L (2016). M1 ipRGCs Influence Visual Function through Retrograde Signaling in the Retina. J Neurosci.

[188] Gamlin PDR, McDougal DH, Pokorny J, Smith VC, Yau K-W, Dacey DM (2007). Human and macaque pupil responses driven by melanopsin-containing retinal ganglion cells. Vision Res.

[189] Kawasaki A, Herbst K, Sander B, Milea D (2010). Selective wavelength pupillometry in Leber hereditary optic neuropathy. Clin Exp Ophthalmol.

[190] Brown TM, Brainard GC, Cajochen C, Czeisler CA, Hanifin JP, Lockley SW (2022). Recommendations for daytime, evening, and nighttime indoor light exposure to best support physiology, sleep, and wakefulness in healthy adults. PLoS Biol.

[191] Huang X, Tao Q, Ren C (2024). A Comprehensive Overview of the Neural Mechanisms of Light Therapy. Neurosci Bull.

[192] Obayashi K, Saeki K, Iwamoto J, Ikada Y, Kurumatani N (2013). Exposure to light at night and risk of depression in the elderly. J Affect Disord.

[193] Wang F, So K-F, Xiao J, Wang H (2021). Organ-organ communication: the liver's perspective. Theranostics.

[194] Toljan K, Homolak J (2021). Circadian changes in Alzheimer's disease: Neurobiology, clinical problems, and therapeutic opportunities. Handb Clin Neurol.

[195] Zuzuárregui JRP, During EH (2020). Sleep Issues in Parkinson's Disease and Their Management. Neurotherapeutics.

[196] Lin M-S, Liao P-Y, Chen H-M, Chang C-P, Chen S-K, Chern Y (2019). Degeneration of ipRGCs in Mouse Models of Huntington's Disease Disrupts Non-Image-Forming Behaviors Before Motor Impairment. J Neurosci.

[197] Adhikari P, Zele AJ, Thomas R, Feigl B (2016). Quadrant Field Pupillometry Detects Melanopsin Dysfunction in Glaucoma Suspects and Early Glaucoma. Sci Rep.

[198] Peng X, Fan R, Xie L, Shi X, Dong K, Zhang S (2022). A Growing Link between Circadian Rhythms, Type 2 Diabetes Mellitus and Alzheimer's Disease. Int J Mol Sci.

[199] Wu J, Duan C, Yang Y, Wang Z, Tan C, Han C (2023). Insights into the liver-eyes connections, from epidemiological, mechanical studies to clinical translation. J Transl Med.

[200] Simats A, Zhang S, Messerer D, Chong F, Beşkardeş S, Chivukula AS (2024). Innate immune memory after brain injury drives inflammatory cardiac dysfunction. Cell.

